# Evaluation of the association of heterozygous germline variants in *NTHL1* with breast cancer predisposition: an international multi-center study of 47,180 subjects

**DOI:** 10.1038/s41523-021-00255-3

**Published:** 2021-05-12

**Authors:** Na Li, Magnus Zethoven, Simone McInerny, Lisa Devereux, Yu-Kuan Huang, Niko Thio, Dane Cheasley, Sara Gutiérrez-Enríquez, Alejandro Moles-Fernández, Orland Diez, Tu Nguyen-Dumont, Melissa C. Southey, John L. Hopper, Jacques Simard, Martine Dumont, Penny Soucy, Alfons Meindl, Rita Schmutzler, Marjanka K. Schmidt, Muriel A. Adank, Irene L. Andrulis, Eric Hahnen, Christoph Engel, Fabienne Lesueur, Elodie Girard, Susan L. Neuhausen, Elad Ziv, Jamie Allen, Douglas F. Easton, Rodney J. Scott, Kylie L. Gorringe, Paul A. James, Ian G. Campbell

**Affiliations:** 1 Cancer Genetics Laboratory, Peter MacCallum Cancer Centre, Melbourne, Vic Australia; 2 Sir Peter MacCallum Department of Oncology, University of Melbourne, Melbourne, Vic Australia; 3 Parkville Familial Cancer Centre, Peter MacCallum Cancer Centre and Royal Melbourne Hospital, Melbourne, Vic Australia; 4 Bioinformatics Core Facility, Peter MacCallum Cancer Centre, Melbourne, Vic Australia; 5 Lifepool, Peter MacCallum Cancer Centre, Melbourne, Vic Australia; 6 Upper Gastrointestinal Translational Research Laboratory, Peter MacCallum Cancer Centre, Melbourne, Vic Australia; 7 Department of Medicine, Royal Melbourne Hospital, The University of Melbourne, Melbourne, Vic Australia; 8 Hereditary Cancer Genetics Group, Vall d’Hebron Institute of Oncology (VHIO); Vall d’Hebron Barcelona Hospital Campus, Barcelona, Spain; 9 Area of Clinical and Molecular Genetics, Hospital Universitari Vall d’Hebron, Vall d’Hebron Barcelona Hospital Campus, Barcelona, Spain; 10 Precision Medicine, School of Clinical Sciences at Monash Health, Monash University, Clayton, Australia; 11 Department of Clinical Pathology, University of Melbourne, Melbourne, Victoria, Australia; 12 Centre for Epidemiology and Biostatistics, Melbourne School of Population and Global Health, The University of Melbourne, Melbourne, Victoria, Australia; 13 Genomics Center, Centre Hospitalier Universitaire de Québec – Université Laval Research Center, Quebec, Canada; 14 University of Munich, Campus Großhadern, Department of Gynecology and Obstetrics, Munich, Germany; 15 Faculty of Medicine and University Hospital Cologne, University of Cologne, Center for Familial Breast and Ovarian Cancer, Cologne, Germany; 16 Faculty of Medicine and University Hospital Cologne, University of Cologne, Center for Integrated Oncology (CIO), Cologne, Germany; 17 Faculty of Medicine and University Hospital Cologne, University of Cologne, Center for Molecular Medicine Cologne (CMMC), Cologne, Germany; 18 Division of Molecular Pathology, The Netherlands Cancer Institute - Antoni van Leeuwenhoek Hospital, Amsterdam, The Netherlands; 19 Division of Psychosocial Research and Epidemiology, The Netherlands Cancer Institute - Antoni van Leeuwenhoek hospital, Amsterdam, The Netherlands; 20 Family Cancer Clinic, The Netherlands Cancer Institute, Amsterdam, The Netherlands; 21 Lunenfeld-Tanenbaum Research Institute, Sinai Health System, Toronto, ON Canada; 22 Department of Molecular Genetics, University of Toronto, Toronto, ON Canada; 23 Leipzig Research Centre for Civilization Diseases, University of Leipzig, Leipzig, Germany; 24 Institute for Medical Informatics, Statistics and Epidemiology, University of Leipzig, Leipzig, Germany; 25 Inserm, U900, Institut Curie, PSL University, Mines ParisTech, Paris, France; 26 Department of Population Sciences, Beckman Research Institute of City of Hope, Duarte, CA USA; 27 Department of Medicine, University of California San Francisco Helen Diller Family Comprehensive Cancer Center, San Francisco, CA USA; 28 Centre for Cancer Genetic Epidemiology, Department of Public Health and Primary Care, University of Cambridge, Cambridge, UK; 29 Centre for Cancer Genetic Epidemiology, Department of Oncology, University of Cambridge, Cambridge, UK; 30 School of Biomedical Sciences and Pharmacy, University of Newcastle, Callaghan, NSW Australia; 31 Discipline of Medical Genetics, The University of Newcastle and Hunter Medical Research Institute, Newcastle, NSW Australia; 32 Division of Molecular Medicine, Pathology North, Newcastle, NSW Australia; 33 Cancer Genomics Program, Peter MacCallum Cancer Centre, Melbourne, Vic Australia

**Keywords:** Cancer genomics, Mutation, Cancer genetics, Breast cancer, Gene expression

## Abstract

Bi-allelic *loss-of-function* (LoF) variants in the base excision repair (BER) gene *NTHL1* cause a high-risk hereditary multi-tumor syndrome that includes breast cancer, but the contribution of heterozygous variants to hereditary breast cancer is unknown. An analysis of 4985 women with breast cancer, enriched for familial features, and 4786 cancer-free women revealed significant enrichment for *NTHL1* LoF variants. Immunohistochemistry confirmed reduced NTHL1 expression in tumors from heterozygous carriers but the *NTHL1* bi-allelic loss characteristic mutational signature (SBS 30) was not present. The analysis was extended to 27,421 breast cancer cases and 19,759 controls from 10 international studies revealing 138 cases and 93 controls with a heterozygous LoF variant (OR 1.06, 95% CI: 0.82–1.39) and 316 cases and 179 controls with a missense variant (OR 1.31, 95% CI: 1.09–1.57). Missense variants selected for deleterious features by a number of in silico bioinformatic prediction tools or located within the endonuclease III functional domain showed a stronger association with breast cancer. Somatic sequencing of breast cancers from carriers indicated that the risk associated with *NTHL1* appears to operate through haploinsufficiency, consistent with other described low-penetrance breast cancer genes. Data from this very large international multicenter study suggests that heterozygous pathogenic germline coding variants in *NTHL1* may be associated with low- to moderate- increased risk of breast cancer.

## Introduction

*NTHL1* encodes a DNA glycosylase that is a critical component of the DNA base excision repair (BER) pathway involved in the repair of oxidatively damaged DNA. It has recently been shown that carriers of bi-allelic *loss-of-function* (LoF) variants in *NTHL1* are predisposed to colorectal adenomatous polyposis and colorectal cancer^1^, and to a multi-tumor syndrome that includes a high incidence of breast cancer in female carriers^2–5^. Grolleman et al.^4^ described the largest set of carriers of bi-allelic germline *NTHL1* variants (29 carriers from 17 families), and reported that 9 of 15 female carriers (60%) were diagnosed with breast cancer at an earlier age than observed in the general population (48.5 years compared to 62 years). In contrast to the previously described BER defect caused by MUYTH deficiency, multiple tumor types from carriers of germline bi-allelic *NTHL1* LoF variants exhibit a distinctive somatic mutation pattern (Single Base Substitution Signature 30 [SBS30] in the COSMIC database^1,6^) characterized by an abundance of C > T transitions at non-CpG sites, indicating that an *NTHL1*-driven BER defect was the predominant mutational process driving the development of these tumors. These data demonstrated that germline bi-allelic inactivation of *NTHL1* predisposes to breast cancer, although individuals with two LoF variants are very rare in the population^4^. In contrast, 0.37% of non-cancer participants in gnomAD are carriers of monoallelic LoF variants (gnomAD V2.1.1, 134,187 participants), but whether carriers are also predisposed to breast cancer has not been evaluated. To address this question, this study sequenced all exons and exon-intron boundaries of *NTHL1* in 9,771 subjects in the hereditary BrEAst Case CONtrol (BEACCON) study, comprising index cases from hereditary breast cancer families who tested negative for germline pathogenic variants in *BRCA1 and BRCA2* and cancer-free older female controls (average 49.7 years vs. 65.6 years) in the same population. In addition, whole-genome and targeted sequencing was performed on formalin-fixed, paraffin-embedded (FFPE)-derived DNA from the breast cancers of 20 germline *NTHL1* LoF variant carriers. Further *NTHL1* sequencing data were analyzed from nine additional case–control studies, to give a combined analysis of 47,180 subjects including 27,421 cases and 19,759 controls.

## Results

### Germline variants in NTHL1 are associated with breast cancer susceptibility in the BEACCON hereditary case–control study

All exons and exon-intron boundaries of the *NTHL1* gene were sequenced in the BEACCON study of index cases from 4985 hereditary breast cancer families and 4786 cancer-free female controls from the same Australian population. A total of eight unique LoF variants were identified among 39 cases and 15 controls (0.78% vs. 0.31%, odds ratio [OR] 2.51, 95% CI: 1.35–4.90, *P* = 0.002) (Table 1). p.(Gln90Ter) was the most frequent variant accounting for 25 (0.50%) cases and 11 (0.23%) controls, followed by p.(Gln287Ter) accounting for 9 (0.18%) cases and 2 (0.04%) controls. The observed frequency of these two variants in the controls were consistent with the carrier frequency reported among 134,187 non-cancer subjects in the gnomAD database (0.29% and 0.03%, respectively; database version v2.1.1). A single individual, homozygous for p.(Gln90Ter) variant, was the only bi-allelic carrier identified; a case subject with a personal history of multiple primary cancers including bilateral breast cancer and colorectal cancer, consistent with the previously reported syndrome for bi-allelic LoF of *NTHL1*^1^ (Supplementary Table 1, C21552; pedigree data for this individual was published previously^4^). In contrast, the heterozygous case carriers were predominantly only affected with breast cancer (Supplementary Table 1). A case–case analysis of cancer incidence in the families of individuals harboring heterozygous *NTHL1* LoF variants found no statistically significantly elevated incidence of colorectal cancer, female breast cancer, male breast cancer or ovarian cancer when compared to the *NTHL1* wild-type families, although the number of available *NTHL1* families was small and the statistical power was limited (Table 2).

**Table 1:** *NTHL1* LoF variants identified in familial breast cancer cases and cancer-free controls in BEACCON study.

Consequence	Case *n* = 4985	Control *n* = 4786	Nucleotide change^a^	Protein change^b^	Exon (of 6)	dbSNP ID	GnomAD^c^
Frameshift	1	0	c.64_83delAGCCTGGGACCCGGGGCTGG	p.Ser22AlafsTer5	1	–	0
Stop Gained	25^d^	11	c.268 C > T	p.Gln90Ter	2	rs150766139	1.44 × 10^−3^
Frameshift	1	0	c.380_383dupTACG	p.Arg129ThrfsTer42	3	rs566860680	4.48 × 10^−6^
Stop Gained	0	1	c.390 C > A	p.Tyr130Ter	3	rs371328106	2.36 × 10^−5^
Stop Gained	0	1	c.390 C > G	p.Tyr130Ter	3	–	0
Stop Gained	2	0	c.457 C > T	p.Arg153Ter	3	rs374489979	1.28 × 10^−5^
Stop Gained	1	0	c.760 A > T	p.Lys254Ter	5	–	0
Stop Gained	9	2	c.859 C > T	p.Gln287Ter	6	rs146347092	1.62 × 10^−4^
Total	39	15	–	–	–	–	–

^a^ENST00000219066.1(NM_002528.5).

^b^ENSP00000219066.1(NP_002519.1).

^c^gnomAD, the minor allele frequency of each variant in 134,187 samples from non-cancer cohorts in GnomAD database V2.1.1.

^d^Including one homozygous carrier.

**Table 2:** Incidence of other cancers in *NTHL1* families compared to non-*NTHL1* families in BEACCON study.

Family history^a^	NTHL1 families^b^ *n* = 22		Non*-*NTHL1 families^c^ *n* = 3239		OR	95% CI	*p* Value
	Cancer (%)	Non-cancer	Cancer (%)	Non-cancer			
Breast cancer	28 (6.93)	376	3929 (6.46)	56,872	1.08	0.71–1.59	0.68
Male breast cancer	1 (0.25)	403	77 (0.13)	60,724	1.96	0.05–11.29	0.40
Colorectal cancer	9 (2.23)	395	1559 (2.56)	59,242	0.87	0.39–1.66	0.87
Ovarian cancer	6 (1.49)	398	541 (0.89)	60,260	1.68	0.61–3.71	0.18

^a^Cancer affected family members in the first and second degree of relatives of index cases.

^b^*NTHL1* families, families in which the index cases carry a germline monoallelic LoF variant in *NTHL1*.

^c^Non-*NTHL1* families, families in which the index cases do not carry any LoF variants in *NTHL1*.

Missense variants in *NTHL1* were also significantly enriched in the cases compared to the controls (75, 1.50% vs. 47, 0.98%, OR 1.54, 95% CI: 1.05–2.27, *P* = 0.02). All the variants were individually “rare” with the highest minor allele frequency [MAF] detected in the controls being 0.0015 in BEACCON and 0.0018 in gnomAD (Supplementary Table 2). Consistent with the frequencies reported in gnomAD, p.(Arg100Cys) and p.(IIe176Thr) were the most common missense variants in both the case and control cohorts. The association of *NTHL1* missense variants with hereditary breast cancer remained when applying a population rarity filter or in silico prediction tools, Condel, PolyPhen2, SIFT, CADD, and REVEL to enrich for likely pathogenic variants, with the strongest effect observed for the missense variants that were selected for rarity (MAF ≤ 0.001, 39 versus 20, OR 1.88, 95% CI: 1.07–3.41) (Supplementary Table 3).

In contrast to the high penetrance reported for the multi-tumor syndrome associated with bi-allelic LoF in *NTHL1*^4^, the OR for heterozygous LoF or missense variants suggests only a moderate- to low-penetrance effect. In this context, the background genetic risk contributed by common, low-penetrance single nucleotide polymorphisms (SNPs) may have an important risk-modifying effect as described for other low-moderate penetrance genes^7^. To assess this possibility, 70 SNPs with well-established, significant associations with breast cancer were used to calculate a polygenic risk score (PRS) for each carrier as described previously^8^. A multivariable logistic regression model was used to simultaneously evaluate the association of *NTHL1* LoF variants, missense variants, and the PRS with breast cancer in the case–control cohort. The ORs observed for LoF variants, missense variants, and the OR per unit standard deviation for the PRS were 2.41 (95% CI: 1.30–4.44, *P* = 0.005), 1.57 (95% CI: 1.09–2.28, *P* = 0.02) and 1.56 (95% CI: 1.50–1.63, *P* < 0.001), respectively. The ORs for LoF and missense variants in *NTHL1* were not attenuated by the inclusion of PRS in the model indicating that the risk associated with *NTHL1* coding variants is independent of any familial aggregation of polygenic risk that may be present in this cohort due to the ascertainment based on family history. When the effect of the PRS and *NTHL1* status was considered in combination the OR for *NTHL1* carriers in the highest 20% of PRS was 3.88 (95% CI: 1.25–15.97, *P* = 0.012) for the LoF variants and 3.03 (95% CI: 1.44–6.90, *P* = 0.001) for the missense variants. This result indicates that although the measured level of risk associated with potentially pathogenic *NTHL1* variants in isolation is below the current threshold for clinical intervention, a proportion of *NTHL1* germline variant carriers in the highest PRS quintile reach a clinically actionable level, as has been described for other low-moderate breast cancer genes^7–9^. The distribution of *NTHL1* LoF and missense variant carriers by PRS quintile (relative to the controls in the BEACCON study) is shown in Supplementary Fig. 1.

### Co-segregation analysis in families with germline NTHL1 variants

Co-segregation analysis of breast cancer in families was performed in 16 multi-case families from the BEACCON study segregating *NTHL1* LoF variants where detailed pedigree information was available. Sanger sequencing was performed to determine the genotype of 38 additional family members. Analysis of co-segregation using a full-likelihood method^10^ calculated a maximum likelihood ratio of 1.18 at an odds ratio for breast cancer of 1.69—insufficient to either support or reject an association with breast cancer predisposition.

### Evaluation of bi-allelic inactivation in NTHL1 associated breast cancers

Histopathologic characteristics are summarized for 22 breast cancers from 20 *NTHL1* LoF variant carrier cases in the BEACCON study where pathology information was available (Supplementary Table 4). The individual with a homozygous p.(Gln90Ter) variant had bilateral breast cancer at age 47 (grade 2, invasive lobular carcinoma, ER+, PR+, HER2−) and 53 (grade 2, invasive ductal carcinoma, ER+, PR+, HER2−). The 21 breast cancers from 19 heterozygous LoF variant carrier cases were predominantly high-grade, invasive, ductal carcinomas (19/21) and hormone receptor-positive (16/21) of which three had *ERBB2* amplification (HER2+).

**Fig. 1:**
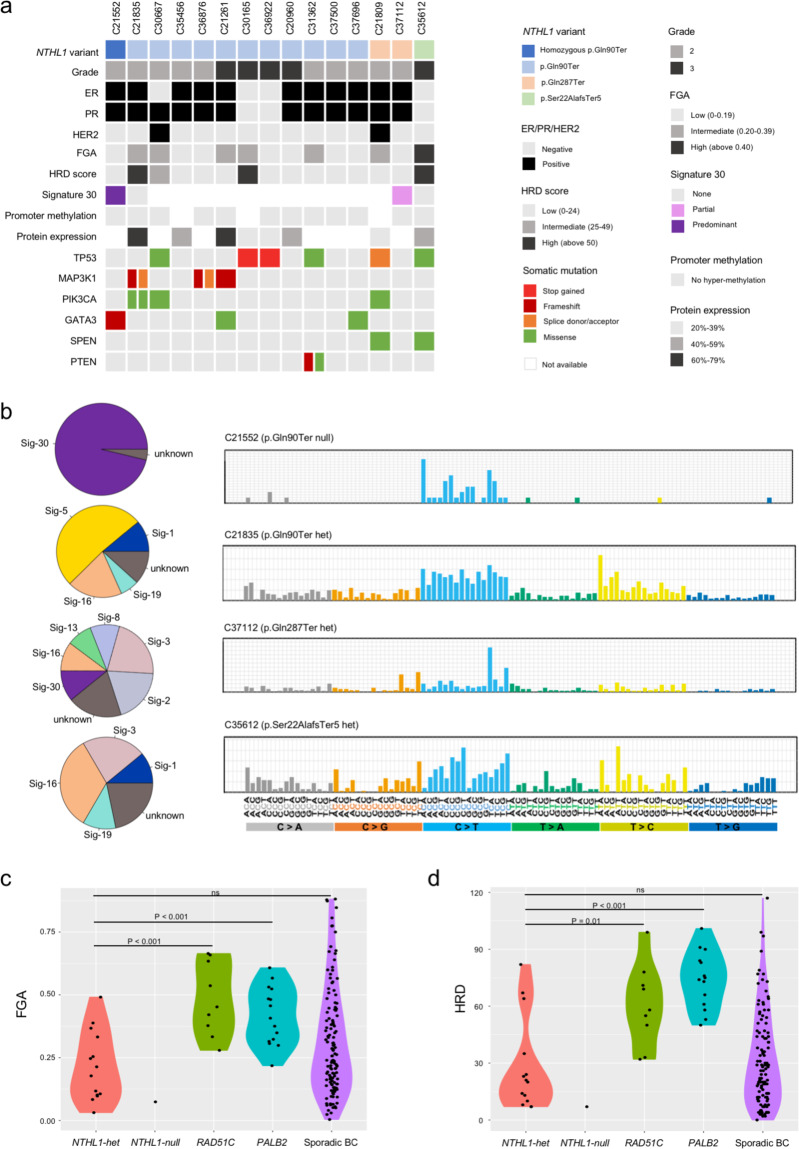
Genomic characterization of breast and ovarian cancers from carriers of *NTHL1* germline loss-of-function variants. **a** Germline variants, somatic mutations and HRD score of *NTHL1* associated tumors. Germline and somatic variant types are color-coded according to the legend. The phenobar provides information about estrogen receptor (ER), progesterone receptors (PR) and human epidermal growth factor receptor 2 (HER2) status, and somatic mutations in driver genes. **b** The weighted contribution of mutational signatures from breast cancers of *NTHL1* germline variant carriers. **c** Fraction of genome altered (FGA) and **d** Homologous recombination deficiency score (HRD) for *NTHL1*-null (*n* = 1) and *NTHL1*-het (*n* = 14) breast cancers compared to breast cancers from *PALB2* (*n* = 15) and *RAD51C* (*n* = 9) germline LoF variant carriers, and sporadic breast cancers (*n* = 115).

To assess the occurrence of somatic bi-allelic inactivation in *NTHL1* and characteristic mutational signatures in *NTHL1*-associated tumors, targeted sequencing was performed on tumor DNA from cases with a germline *NTHL1* LoF variant using a custom-designed panel of 259 genes (total targeted region of 1.337 Mb) that included all exons and exon-intron boundaries of *NTHL1* and 27 breast-cancer driver genes^11^. Fourteen breast cancers and ovarian cancer from individuals with heterozygous *NTHL1* germline variants, together with breast cancer and colorectal cancer from the individual homozygous for germline LoF variants, were sequenced. The alternative allele frequency of the germline variants, reconstructed copy number profile, and tumor purity estimation was used to determine loss of heterozygosity in the *NHTL1*-associated tumors as described previously^12,13^. The bi-allelic mutation was confirmed in the breast and colorectal cancers from the homozygous carrier, however, there was no evidence of loss of the wild type allele or a second point mutation in *NTHL1* in any cancers from heterozygous germline variant carriers. Since CpG island methylation in the promoter region is a possible alternative mechanism of gene silencing, bisulfite sequencing was performed across the *NTHL1* promoter region for 13 breast cancers with sufficient DNA available. No evidence of hypermethylation was observed in any of the cancers tested (Fig. 1a, Supplementary Fig. 2).

### Mutational signatures in NTHL1 associated breast cancers

Targeted sequencing in 14 breast cancers from individuals harboring an *NTHL1* heterozygous variant (*NTHL1-het*) identified somatic mutations in breast cancer driver genes including *TP53* (6/14 cases), *PIK3CA* (3/14 cases), *MAP3K1* (3/14 cases), and *GATA3* (2/14 cases) (Fig. 1a). This spectrum of somatic mutations in *NTHL1-het* tumors was similar to that observed in sporadic breast cancer, with mutations in *TP53* and *PIK3CA* the most common, and mutations in *MAP3K1* and *GATA3* frequently occurring in ER+ cancers^14^.

To investigate whether *NTHL1*-associated breast cancers are driven by the same mutagenesis mechanism as the colorectal cancers from carriers of homozygous mutations^1,6^, whole-genome sequencing was performed on the breast cancers from the *NTHL1-null* and *NTHL1-het* carriers of three different germlines LoF variants (p.(Gln90Ter), p.(Gln287Ter), and p.(Ser22AlafsTer5)), together with nine sporadic breast cancers with no known germline cancer predisposition gene mutations as controls. A predominant mutational signature SBS30 was observed in the *NTHL1-null* breast cancer (Fig. 1b), consistent with the previously reported *NTHL1* bi-allelic loss driving tumorigenesis mechanism. In line with the absence of bi-allelic inactivation, the three *NTHL1-het* breast cancers each exhibited a mixture of mutational signatures, with the top contributing signatures including COSMIC signatures SBS3, SBS5, and SBS16, similar to the sporadic breast cancers. While one of the three *NTHL1-het* cancers showed a minor proportion of SBS30 (C37112, p.(Gln287Ter), Fig. 1b), this was also observed in one of the nine sporadic cancers, suggesting no major difference in mutational processes between *NTHL1-het* and sporadic control cancers (BC3, Supplementary Fig. 3).

### Fraction of genome alteration and HRD scores in NTHL1 associated breast cancers

Genomic instability and HRD were measured using genome-wide copy number data from the *NTHL1* associated cancers. A genomic instability index, a fraction of the genome altered by copy number (FGA)^15^, and an HRD score^16,17^ were generated for 14 *NTHL1-het* cancers and one *NTHL1-null* cancer. A comparison cohort of breast cancers with expected HR defects from *PALB2* (*n* = 15) and *RAD51C* (*n* = 9) germline LoF variant carriers, and 115 sporadic breast cancers sequenced using the same platform, were also evaluated. The *NTHL1-het* cancers exhibited a broad range of FGA scores (Fig. 1c) that were significantly lower than the *PALB2* associated cancers (median 0.20 vs. 0.41, *P* < 0.001 by Mann-Whitney test) or the *RAD51C* associated cancers (median 0.20 versus 0.45, *P* < 0.001) and not statistically significantly different to the FGA scores observed in the sporadic breast cancers (median 0.20 vs. 0.26, *P* = 0.11). Similarly, the HRD scores for *NTHL1*-het cancers were significantly lower than those observed in the *PALB2* associated cancers (median 21.5 vs. 74.5, *P* < 0.001) or the *RAD51C* associated cancers (median 21.5 vs. 58, *P* = 0.01), and not significantly different to those observed in sporadic cancers (median 21.5 vs. 27, *P* = 0.79) (Fig. 1d). The one *NTHL1-null* breast cancer also showed a very low FGA and HRD score (0.07 and 7).

### NTHL1 protein expression in NTHL1 associated breast cancers

**Fig. 2:**
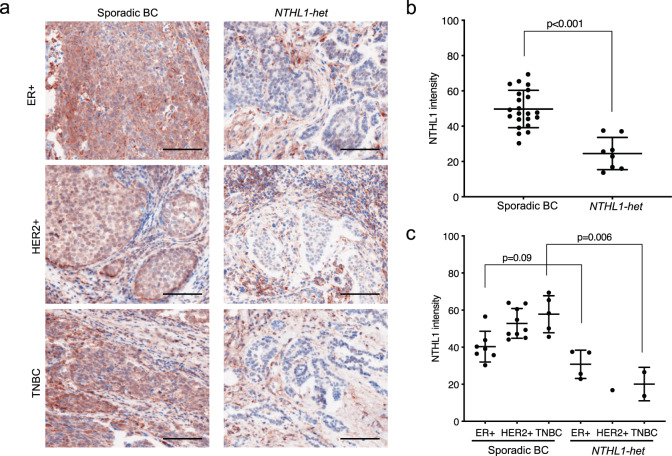
NTHL1 protein expression in sporadic breast cancer (*n* = 21) and *NTHL1*-het breast cancer (*n* = 8). **a** NTHL1 expression in sporadic breast cancer and *NTHL1*-het cancer of ER+, HER2+, and triple-negative types. Multiplex immunofluorescent staining approach was used and the fluorescence signal was displayed in colorimetric pattern for better contrast. NTHL1: brown color; DIPI: blue color. The epithelial marker AE1/AE3 (Supplementary Fig. 4) was used to identify cancer cells in the breast cancer tissue in NTHL1 expression quantitation in (**b**) and (**c**). Scale bar = 100 μm. **b** The average intensity of NTHL1 in sporadic cancer group compared to NTHL1-het group. **c** The average intensity of NTHL1 in sporadic cancer group compared to *NTHL1*-het group according to ER, PR, and HER2 status. BC breast cancer, TNBC triple-negative breast cancer.

To investigate whether heterozygous *NTHL1* LoF variants were associated with reduced protein expression and/or altered cellular location in breast cancers as has been observed in gastric tumors^18^, fluorescent immunohistochemistry was used to measure NTHL1 protein levels, along with an epithelial cell marker (Cytokeratin AE1/AE3), in 8 *NTHL1-het* breast cancers and 21 sporadic breast cancers. NTHL1 had a predominantly nuclear localization in both wild-type and *NTHL1*-het cancers. Compared to sporadic breast cancers, *NTHL1-het* cancers as a group showed a 51% reduction in NTHL1 staining in the AE1/AE3-positive cancer cells (average staining intensity 24.51 vs. 49.83, *P* < 0.001 by unpaired *t* test) (Fig. 2a, b) and a 40% reduction in the AE1/AE3-negative non-cancer cells (35.62 vs. 59.65, *P* < 0.001). In addition, for 5 of the 8 *NTHL1-het* cancers, the expression of NTHL1 in the breast cancer cells (AE1/AE3 positive cells) was reduced by >30% compared to the surrounding non-cancer cells (AE1/AE3 negative cells) (Supplementary Fig. 4), while only a small proportion of control cancers showed the same phenomenon (5 of 21), suggesting that NTHL1 expression may be attenuated further specifically in the cancer cells of *NTHL1* LoF variant carriers. When considering the pathological subtypes individually the average NTHL1 expression level was lower in *NTHL1-het* tumors than in the controls in all three major subtypes: ER+, HER2+, and triple-negative cancers, but only reached statistical significance in the triple-negative cancers (Fig. 2c).

### Analysis of NTHL1 in multi-center international case–control cohorts

To evaluate the role of *NTHL1* in breast cancer predisposition in diverse populations and independent studies, a total of 27,421 cases and 19,759 controls were screened for the entire coding region of *NTHL1* from ten case–control studies including BEACCON as discovery dataset, and nine additional studies as validation dataset. The validation dataset included SEARCH, UK Population-based Breast Cancer Study; GC-HBOC, German Consortium for Hereditary Breast and Ovarian Cancer; GENESIS, French familial BRCAx study (some data were previously published^19^); VHIO, familial breast cancer, and control study of the Vall d’Hebron Institute of Oncology of Barcelona; OFBCR, Ontario Familial Breast Cancer Registry; DFBBCS, the Dutch Familial Bilateral Breast Cancer Study; HABC, Hispanic–American Breast Cancer Study; ABCFR, Australian Breast Cancer Family Registry; and CARTaGENE, Québec Population-based Breast Cancer Study. The information of cohorts and subjects, sequencing platform, and coverage are summarized in Supplementary Table 5.

**Fig. 3:**
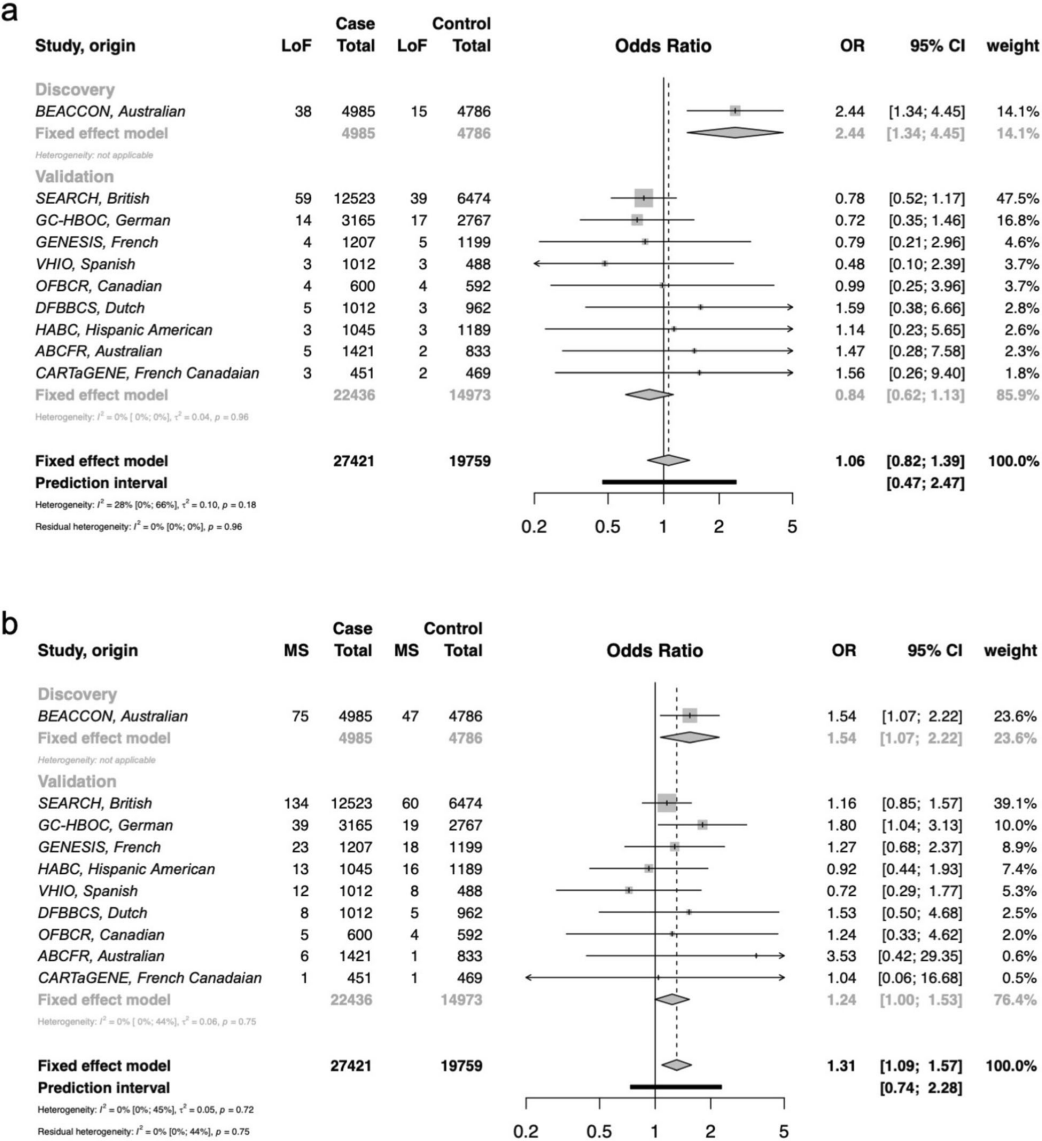
Frequency of heterozygous germline variants in *NTHL1* and odds ratios for breast cancer observed in case–control data from ten multicenter international cohorts. **a** Heterozygous loss-of-function variants in *NTHL1* and odds ratios in ten case–control cohorts. **b** Heterozygous missense variants in *NTHL1* and odds ratios in ten case–control cohorts. The overall effect of odds ratios were computed based on a fixed-effect model. BEACCON hereditary BrEAst Case CONtrol study, SEARCH UK Population-based Breast Cancer Study, GC-HBOC German Consortium for Hereditary Breast and Ovarian Cancer, GENESIS French familial BRCAx study (some data were previously published^19^), VHIO familial breast cancer and control study of the Vall d’Hebron Institute of Oncology of Barcelona, OFBCR Ontario Familial Breast Cancer Registry, DFBBCS the Dutch Familial Bilateral Breast Cancer Study, HABC Hispanic-American Breast Cancer Study, ABCFR Australian Breast Cancer Family Registry, CARTaGENE Québec Population-based Breast Cancer Study.

**Fig. 4:**
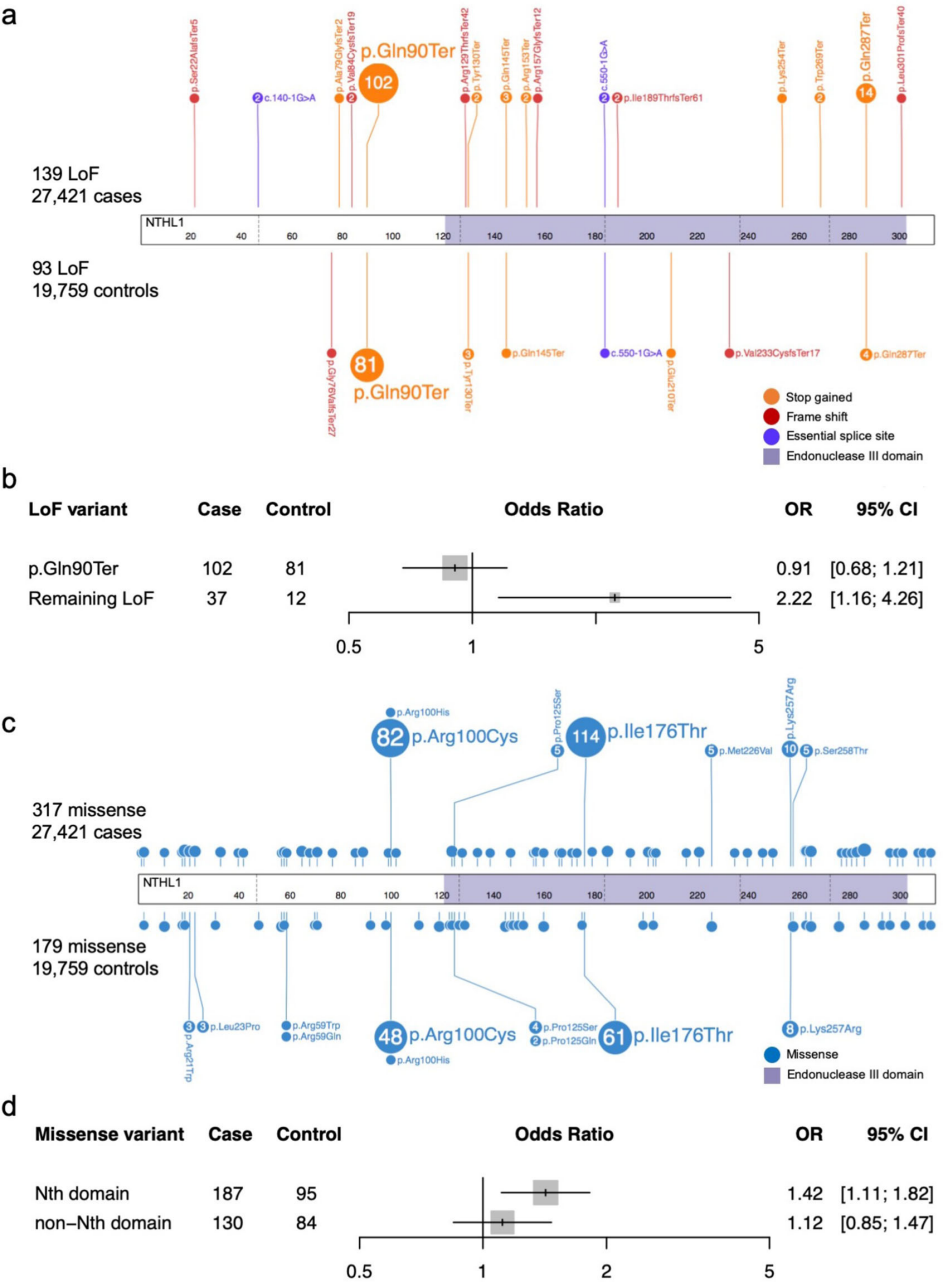
Germline *NTHL1* variants identified in 27,421 cases and 19,759 controls. **a** Lollipop plot of all LoF variants identified in 27,421 cases and 19,759 controls. **b** Odds ratio (OR) and 95% confidence interval (95% CI) for recurrent LoF variants p.(Gln90Ter) and all the rest of rare LoF variants (MAF < 0.001 according to gnomAD). **c** Lollipop plot of all missense variants identified in 27,421 cases and 19,759 controls. Nth, Endonuclease III. **d** OR and 95% CI for missense variants located in functional domain and the rest of the NTHL1 protein.

No additional bi-allelic LoF variants were identified in subjects of the validation dataset, indicating germline bi-allelic loss of *NTHL1* is extremely rare as a cause of breast cancer (1/27,421 breast cancer cases and 0/19,759 controls). In the heterozygous state, the overall effect observed in the validation dataset (OR = 0.84, 95%CI = 0.62-1.13) did not support the finding in the BEACCON dataset, with 4/9 studies showing a weak positive association while the others showed no effect or a weak negative association, although the sample sizes of most studies were small (Fig. 3a). The frequency of LoF variants observed in each study varied greatly among both the cases (0.29–0.76%) and the controls (0.24–0.68%), largely driven by differences in frequency of the predominant variant p.(Gln90Ter) (Fig. 4a; Supplementary Table 6). The remaining LoF variants identified were all rare in gnomAD (MAF < 0.001), with an overall statistically significant enrichment, observed in the cases in the combined data for these variants from all 10 studies (37 vs. 12, OR 2.22, 95% CI: 1.16–4.26, Fig. 4b).

In contrast to the LoF variants, the frequency of missense variants was higher among the cases in the majority of studies in the validation dataset (6/9 studies), and was statistically significant in the overall analysis of the validation dataset (OR 1.24, 95% CI: 1.00–1.53) and the combined BEACCON and validation datasets (OR 1.31, 95% CI: 1.09–1.57) (Fig. 3b). While the detected missense variants were distributed across the whole gene (Supplementary Table 6), there was an enrichment of missense variants in the cases compared to controls in the Endonuclease III domain of *NTHL1* (187 vs. 95, OR 1.42, 95% CI: 1.11–1.82) (Fig. 4c, d). When a number of in silico prediction tools and population rarity filters were applied to enrich for likely pathogenic variants in the combined data from the ten studies, the predicted missense variants were observed in excess in the cases compared to the controls and reached statistical significance in all the in silico prediction groups: Condel, PolyPhen2, SIFT, CADD, and REVEL (Table 3). The strongest enrichment was observed for variants with a high REVEL score of pathogenicity (REVEL > 0.75, OR 1.44, 95% CI: 1.08–1.94), and interestingly these likely pathogenic variants according to REVEL were also predominantly located in the conserved functional domain of NTHL1 protein, Endonuclease III (COG0177, a member of ENDO3c Superfamily) (Supplementary Fig. 5).

**Table 3:** Likely pathogenic missense variants of *NTHL1* selected by population rarity or deleterious in silico tool predictions in case–control data of ten multi-center international cohorts.

NTHL1 missense variants	Case *n* = 27,421		Control *n* = 19,759		OR	95% CI	*p* Value
	Carriers	%	Carriers	%			
Total^a^	316	1.15	179	0.91	1.28	1.06–1.54	0.01
Rare (MAF ≤ 0.001)^b^	121	0.44	70	0.35	1.25	0.92–1.7	0.16
Rare (MAF ≤ 0.0001)^b^	92	0.34	49	0.25	1.35	0.95–1.96	0.09
Condel (deleterious)	175	0.64	92	0.47	1.37	1.06–1.79	0.02
PolyPhen2 (damaging)	161	0.59	83	0.42	1.4	1.07–1.85	0.01
SIFT (deleterious)	268	0.98	146	0.74	1.33	1.08–1.63	0.006
CADD (≥10)	282	1.03	155	0.78	1.31	1.08–1.61	0.006
REVEL (≥0.75)	144	0.53	72	0.36	1.44	1.08–1.94	0.01

^a^Includes all the missense variants identified.

^b^MAF, minor allele frequency in non-cancer non-Finnish Europeans in gnomAD database.

## Discussion

NTHL1 is a DNA glycosylase involved in the earliest steps of the repair of oxidative DNA damage through the BER pathway. Through its endonuclease activity it acts to excise damaged nucleotides, predominantly pyrimidines, and loss of NTHL1 activity would be expected to impact the effectiveness of normal DNA repair. This effect was shown to be clinically significant by the discovery of a specific cancer predisposition syndrome involving complete loss of normal NTHL1 function through bi-allelic pathogenic germline LoF variants. The resulting condition not only demonstrates high risk for a range of different malignancies, but it was possible to show that specific mutational processes arising from the loss of NTHL1-mediated BER dominated the genetic pathology of the resulting cancers, reflected in the distinctive somatic mutational signature, COSMIC signature SBS30. Although the recessive condition has proved to be very rare, the analogy with other tumor suppressor genes raised the possibility that inheritance of a single germline pathogenic variant followed by somatic inactivation of the wildtype allele could achieve the same outcome through a “two-hit” mechanism with the result that heterozygous variants would also be associated with inherited cancer risk. We have examined this possibility specifically in regard to the risk of breast cancer through sequencing of a large number of cases and controls and examination of the somatic landscape of cancers occurring in women carrying a single *NTHL1* LoF variant. The results have shown that germline bi-allelic loss is not a major contributor to breast cancer in the populations studied and there is equally no evidence that heterozygous variants act through a traditional two-hit pathway to cause breast cancer. However, the data do support a possible low-risk effect for at least some heterozygous LoF variants and rare, deleterious missense variants. Our data reflects the recent findings that heterozygous LoF variants in *NTHL1* do not confer any substantial risk for colorectal cancer and do not undergo bi-allelic inactivation^20^.

The difficulty in providing clear evidence of a breast cancer risk association for LoF variants in the cases and controls appears to reflect, in part, a higher degree of heterogeneity among the ten analyzed studies compared to little variability between studies for missense variants. The statistically significant two-fold excess in the BEACCON study was not observed in the validation dataset and this discrepancy was largely driven by the variable frequency of LoF variants in control populations across cohorts which ranged from 0.24 to 0.68%. In particular, the frequency of the p.(Gln90Ter) variant, which accounted for the large majority of LoF variants, was highly variable among the 10 studies, as it is across different ethnic groups reported in gnomAD; ranging from ~1 in 280 in the Finnish population to less than 1 in 3000 in African and Asian populations. Differences in the ethnic background and ascertainment methods among the different studies may explain the diverse results in the sub-studies. This variability was, however, limited to the p.(Gln90Ter) variant. If the analysis of the data from the ten studies in combination was restricted to the remaining rarer LoF variants, a statistically significant two-fold excess was observed in cases compared to the controls (37, 0.13% of 27,421 cases compared to 12, 0.06% of 19,759 controls, OR 2.22, 95% CI: 1.16–4.26). With the exclusion of the most common LoF variant, the numbers are small and the result requires validation in further studies. Stronger evidence for a low-risk predisposition effect was found for missense variants, which were more consistently enriched in cases across sub-studies but also showed further enrichment when selected for features most likely to be associated with disruption of normal gene function: rarity, deleterious *in silico* properties or location in a major functional domain.

Tumor sequencing and promoter methylation analyses demonstrated that *NTHL1* does not undergo bi-allelic inactivation in breast cancers, which is supported by the fact that *NTHL1-het* tumors retain approximately 50% of the wildtype protein expression. Although Drost et al.^6^ reported loss of the wild-type allele in a single breast cancer carrying an *NTHL1* LoF variant (p.(Gln287Ter)), our data suggest this is not a common event in *NTHL1-*associated breast tumors. The reduced, but not complete loss of NTHL1 expression in *NTHL1-het* cancers is consistent with the fact that, while the mutational spectrum of the *NTHL1-null* breast cancer strongly resembled COSMIC mutational signature SBS30, only minor components of SBS30 were observed in one of three *NTHL1-het* tumors examined. Although the total number of tumors analyzed remains small, the *NTHL1-het* tumors appeared similar to sporadic breast cancers in regard to the somatic mutational features, and in other respects (HRD scores, histopathology, and FGA).

The absence of a second hit in *NTHL1* may be a generic feature of low-moderate penetrance alleles. While many high-risk breast cancer genes *BRCA1*^21^, *BRCA2*^22^, *PALB2*^23^, and *RAD51C*^12^ follow the Knudson two-hit model, where a germline mono-allelic variant in the gene is accompanied by somatic loss of the wild-type allele, the limited data currently available for alleles which confer low- (RR 1–2) to moderate- (RR 2–4) risk suggest they do not always undergo a second hit. For example, a second hit rarely occurs in breast cancers from carriers of the lower penetrance *CHEK2* p.(Ile157Thr) variant^24^ or the *BRCA2* p.(Lys3326Ter) variant^22^, while limited reports suggest that it does occur in breast cancers from women carrying germline *CHEK2* LoF variants^24,25^. It is possible that alleles associated with low or moderate risk mediate cancer predisposition through pathways that do not require a second hit, such as haploinsufficiency where reduced protein levels rather than the complete loss are sufficient to increase the risk of disease^24–33^. Reduced NTHL1 expression has been previously described as a feature of specific malignancies^34,35^ and recent reports have found that *NTHL1* missense variants can induce cellular transformation and genomic instability in vitro while retaining normal cellular location and enzymatic function^36,37^, raising the possibility that a non-canonical function may be involved. Alternatively, the distinction between high-risk cancer susceptibility variants that undergo a somatic second hit and low-risk alleles that do not—even where bi-allelic loss appears to convey a clear oncogenic advantage, as demonstrated in the case of *NTHL1* by the recessive cancer syndrome—directly reflects the fact that these alleles are less prone to obtain a second hit leading to a complete loss of the function, and always retain some activity in the tumor.

In summary, this is the first study to investigate the role of heterozygous *NTHL1* LoF and missense variants in breast cancer predisposition, which included 47,180 subjects from ten international case-control studies. The data suggest that *NTHL1* may be associated with a modest increase in breast cancer risk that would not be considered clinically actionable in isolation under current clinical guidelines but could be relevant when combined with additional risk factors. Molecular analyses of breast cancers from carriers indicate that *NTHL1* may be included in the growing list of low-penetrance breast cancer genes that appear to function via haploinsufficiency rather than the bi-allelic inactivation mechanism almost universally observed for high-risk breast cancer predisposition genes.

## Methods

### Case–control Subjects

All exons and exon-intron boundaries of *NTHL1* were analyzed in the index cases of 4985 hereditary breast cancer families and in 4786 cancer-free women in the hereditary BEACCON study. The cases were female breast and/or ovarian-cancer affected patients (>95% breast cancer affected) in the *Variants in Practice (ViP) Study* that were ascertained from the combined Victorian and Tasmanian Familial Cancer Centres, and Pathology North, NSW Health Pathology, Newcastle, Australia. The controls were cancer-free women who were greater than or equal to 40 years old from the same population from the *Lifepool* study as described previously^38^. A hereditary breast cancer family is defined as those assessed by a specialist Familial Cancer Clinic where most of the affected family members meet family history criteria or have individual risk factors that predict a greater than 10% chance of having a *BRCA1* or *BRCA2* pathogenic variant (a detailed guide is in https://www.eviq.org.au/cancer-genetics/adult/genetic-testing-for-heritable-mutations/620-brca1-and-brca2-genetic-testing). All cases tested negative for pathogenic variants in *BRCA1* and *BRCA2* including large scale genome rearrangements. The average breast cancer diagnosis age of the cases was 49.7 years (range 19.0–94.8). The average age of controls was 65.6 years (range 40.0–97.5). The study was approved by the Human Research Ethics Committee at the Peter MacCallum Cancer Centre (Approval # 09/29) and all participating centers. All participants provided informed consent for genetic analysis of their germline DNA.

Nine international case-control studies of diverse countries of origin and sample sizes in which all exons of *NTHL1* gene were analyzed were used as validation cohorts. The studies were; UK Population-based Breast Cancer Study (SEARCH), German Consortium for Hereditary Breast and Ovarian Cancer (GC-HBOC), French familial BRCAx study (GENESIS, some data were previously published^19^), familial breast cancer and control study of the Vall d’Hebron Institute of Oncology of Barcelona (VHIO), Ontario Familial Breast Cancer Registry (OFBCR), the Dutch Familial Bilateral Breast Cancer Study (DFBBCS), Hispanic–American Breast Cancer Study (HABC), Australian Breast Cancer Family Registry (ABCFR), and Québec Population-based Breast Cancer Study (CARTaGENE). The case subjects in SEARCH, ABCFR, and CARTaGENE were recruited in relevant populations without consideration of enrichment for family history (population-based cohort), while the case subjects in the other studies were ascertained using various standards to enrich for high-risk breast cancer patients (hereditary cohort). The case and control subjects in individual cohorts were sequenced using the same platform and had sufficient and comparable sequencing coverage (Supplementary Table 5).

### *NTHL1* sequencing using germline DNA

Germline DNA from the cases in the BEACCON study was obtained from blood and was extracted in clinical laboratories, and the DNA from controls were obtained from blood (87%) and saliva (13%) and were extracted by *Lifepool* researchers. All exons and 10 bp into each exon–intron boundary of the *NTHL1* gene were sequenced using a customized targeted HaloPlex HS Targeted Enrichment Assay panel (Agilent Technologies, Santa Clara, CA) which was designed using Agilent’s SureDesign tool at https://earray.chem.agilent.com/suredesign/. A set of 74 Ancestry Informative Markers (AIMs)^39–41^ was included in the sequencing of 3409 subjects (1747 cases and 1662 controls) to determine the ethnicity background of study subjects. Furthermore, a total of 70 SNPs that were reported to be associated with breast cancer in GWAS studies^8^ were sequenced in all subjects to calculate breast cancer PRSs. Library preparation was performed using the Agilent NGS Bravo automation system (Agilent Technologies) according to the manufacturer’s protocol (Agilent Technologies, HaloPlex HS Target Enrichment System Automation Protocol For Illumina Sequencing. https://www.agilent.com/cs/library/usermanuals/public/G9931-90010.pdf). Sequencing was performed by the Australian Genome Research Facility (North Melbourne, VIC, Australia) on an Illumina Hiseq2500 sequencer. Library pools of 96 samples were sequenced on a HiSeq2500 Genome Analyzer using 100 bp paired-end reads (Illumina, San Diego, CA) with an average read depth target of >250X. The average sequencing depth yield for *NTHL1* gene was 396.13 and 358.64 for the cases and controls, respectively, with 99.32% and 99.19% of the target bases sequenced ≥10 reads.

### Germline DNA sequencing alignment and variant calling

Sequencing data from the BEACCON study were processed, aligned, and analyzed through a pipeline constructed using Seqliner v0.1a (http://bioinformatics.petermac.org/seqliner) by Bioinformatic Core Facility of Peter MacCallum Cancer Centre as described in detail elsewhere^42^. GATK Unified Genotyper v2.4 (Broad Institute, Cambridge, MA)^43^, Haplotype caller^44^, and PLATYPUS^45^ were used for variant calling. ENST00000219066.1(NM_002528.5) and ENSP00000219066.1(NP_002519.1) were used to annotate the variants identified in *NTHL1*. LoF variants were defined as frameshift, stop gained or essential splice site variants. The MAF reported in ExAC and gnomAD databases were used as a population frequency reference for the variants identified.

### Variant filters and validation

Filters were applied to the sequencing data from BEACCON to remove sequencing artifacts and included a quality score over 30, a minimum of 10 reads, and at least 5 reads supporting the alternative alleles and a variant allele proportion of greater than 20%. In addition, all variants included in the analysis had to pass all the internal filters of at least two out of three variant callers GATK, Unified Genotyper, Platypus, and Haplotype caller. Sequencing BAM files were viewed in IGV to manually curate the accuracy of the variant calls, and all the LoF and missense variants detected in *NTHL1* were validated using Sanger sequencing (primer sequences are listed in Supplementary Table 7). In addition, any case or control carrying an LoF variant in *NTHL1* was reviewed for any pathogenic variants in known hereditary breast cancer genes (*CHEK2*, *PALB2*, *ATM*, *TP53*, *CDH1*, *PTEN*, and *STK11*) or LoF variants in proposed breast cancer genes *(RAD51C*, *RAD51D*, *BRIP1*, *BARD1*, *MRE11A*, *RAD50*, and *NBN*). Two case carriers each had a *CHEK2* c.1100delC variant, and one case carrier had a splice acceptor variant c.2071-1 G > A in *NBN*. No pathogenic variants or LoF variants in any proposed breast cancer genes were identified in any of the control carriers.

### Co-segregation analysis in families with germline *NTHL1* variants

Pedigree information for 16 families with an *NTHL1* LoF variant were obtained and Sanger sequencing was performed to determine the genotype for a total of 38 additional family members from these families. A full-likelihood method was used for co-segregation analysis as described previously^10^. A full pedigree likelihood was calculated as a means of assessing the linkage between the variant and disease based on all available genotype information from the family, including any unaffected individuals who have been tested.

### Tumor microdissection and DNA extraction

FFPE breast or ovarian tumor blocks were obtained from carriers of germline variants in the *ViP* study, and cancer cells were collected through needle microdissection under a dissecting microscope. Hematoxylin and eosin (H&E) stained slide was reviewed for each case to identify cancer cell-enriched regions to guide the microdissection and achieve high tumor purity (aimed to achieve >70% tumor cells). Between 15 and 30 slides per block (depending on the size of the tumor and the proportion of cancer cells) of 8–10 μM thickness was needle microdissected, and the DNA of the collected cancer cells was extracted and purified using the QIAamp DNA FFPE Tissue Kit (Qiagen, Valencia, CA, USA). DNA was quantified using Qubit dsDNA high-sensitivity Assay kit (ThermoFisher Scientific, MA, USA). A multiplex polymerase chain reaction (PCR)-based quality-control method reported by van Beers et al.^46^, was used to identify DNA samples with sufficient quality for molecular analysis.

### Whole-genome sequencing and targeted sequencing of tumor DNA

Whole-genome sequencing (30× coverage, 100 bp paired-end) of four tumor-normal pairs was performed on the BGI-Seq platform using 500 ng of DNA extracted from FFPE tumors or from blood. Targeted sequencing of tumor DNA was performed using an Agilent SureSelect XT Custom Panel that targeted all exons of a total of 259 genes (total targeted region of 1.337 Mb) including all the candidate genes in Halo3 design, and an additional 27 breast cancer driver genes^11^. Sequencing libraries were generated using 300 ng tumor DNA and were sequenced on an Illumina Next Seq 500 (75 bp paired-end reads).

### Tumor DNA sequencing alignment and variant calling

Paired-end sequence reads from tumor DNA were aligned to the GRCh37 human reference genome using BWA-MEM^15^. PCR and optical duplicate reads were removed using Picard (http://broadinstitute.github.io/picard/) and then local realignment around indels and base quality score recalibration were performed using the Genome Analysis Tool Kit (GATK). SNP and indel variants were called using GATK Unified Genotyper^43^, Platypus^45^, and Varscan2^47^.

A quality score over 150, a minimum of 10 reads, and an alternate allele frequency of more than 10% were used to rule out sequencing artifacts. In paired tumor-normal sequencing data, the somatic mutations were identified by removing the germline variants. In tumor sequencing data without matched germline data, somatic mutations were identified by applying stringent filters on the population frequency (minor allele frequency, MAF ≤ 0.0001 in ExAC and gnomAD^48^), the frequency in the in-house sequencing cohort (<20% of 166 breast tumors with the exception of variants in *PIK3CA* and *TP53*), and removing the variants identified in germline sequencing data.

### Genome-wide copy number analysis

Genome-wide copy number data were generated from off-target sequencing reads using CopywriteR v2.10 with 50 kb bins^49^. NEXUS Copy Number^TM^ (software v8.0 with build version 9169, BioDiscovery Inc.) was used for CN segmentation using the SNPFASST2 algorithm with default parameters. Copy number gains and losses were called with log_2_ ratio thresholds of >0.2 and < −0.2, respectively.

### Fraction of genome altered (FGA) and homologous recombination deficiency (HRD) scores

Using the genome-wide copy number data, the FGA was calculated with adjustment according to chromosome sizes as described^50,51^. An HRD score, combined from three HRD score components: number of telomeric allelic imbalances^52^, HRD-loss of heterozygosity^53^, and large-scale state transitions^54^ was calculated as described in detail elsewhere^13^.

### Mutational signature analysis

Mutational signatures were performed and plotted using deconstructSigs^55^ package in R v3.3.2^56^ based on the somatic mutations identified by the whole genome or targeted sequencing of the tumors of interest. For each sample, the somatic substitutions were categorized into six basic base substitutions (C > A, C > G, C > T, T > A, T > C, and T > G) and subcategorized into 96 subcategories according to the trinucleotide context. Mutational signatures were determined referring to the COSMIC mutational signature database.

### *NTHL1* promoter methylation analysis

The promoter regions of *NTHL1* were examined for methylation status by Sanger sequencing the promoter region using bisulfite-treated DNA. Tumor DNA was treated by the EpiTect Fast DNA Bisulfite Kit (Qiagen, Valencia, CA, USA) according to the manufacturer’s instructions, followed by PCR amplification and Sanger sequencing of the promoter regions. The primer pairs were designed using the default settings of the Bisulfite Primer Seeker tool (https://www.zymoresearch.eu/bisulfite-primer-seeker, Supplementary Table 7), and/or from previously published study^57^. CpGenome Human Methylated DNA Standard (Millipore, USA) served as a methylation positive control and Female Genomic Reference DNA (Promega, Madison, WI, USA) as a negative control to assess the methylation status of tumor samples.

### NTHL1 protein expression analysis

Opal multiplex fluorescent immunohistochemistry method (PerkinElmer) was used to evaluate the NTHL1 protein expression in cancer and non-cancer cells in FFPE breast cancer tissue. NTHL1 antibody (Abcam, Branford, Connecticut, USA; 1:1000 dilution) and AE1/AE3 antibody (multi-cytokeratin antibody, Leica Biosystems, Wetzlar, Germany; 1:1000 dilution) were used as primary antibodies. HRP-labeled anti-rabbit antibody (PerkinElmer Waltham, Massachusetts, USA; 1:1000 dilution) and anti-mouse antibody (PerkinElmer, Waltham, Massachusetts, USA; 1:1000 dilution) were used as secondary antibodies. Spectral DAPI (PerkinElmer, Waltham, Massachusetts, USA) was used to label the nuclear. The epithelial marker AE1/AE3 was used to identify cancer cells in the breast cancer tissue, and the average intensity of nuclear expression of NTHL1 was quantitated using PerkinElmer Vectra Automated Multispectral Imaging System.

### Statistical analysis

OR and two-tailed *p* value by Fisher’s exact test for the case and control study were calculated in *R*^56^. The conditional Maximum Likelihood Estimate was used for OR. Fisher’s exact test (two-sided) was used for the comparisons of case–control data, and a *p* value of ≤0.05 was considered as statistically significant. PRS was calculated based on 70 low penetrance breast cancer predisposition SNPs following a multiplicative risk model (calculated by the sum of the minor alleles weighted by the per-allele log OR) described by Mavaddat et al.^8^. Mann–Whitney *U* test was performed for FGA and HRD score comparisons between groups of tumors in GraphPad Prism version 7.00 (California, USA). Unpaired *t* test was used for comparing NTHL1 expression in different comparison groups. The meta-analysis for multi-center international cohorts was performed using meta R package^58^ under a fixed-effect model for analysis within subgroups or analyzing all ten cohorts.

### Reporting summary

Further information on research design is available in the Nature Research Reporting Summary linked to this article.

## Supplementary information

Supplementary material

Reporting Summary

## Data Availability

The data generated and analyzed during this study are described in the following data record: 10.6084/m9.figshare.14208293^59^. The sequencing data have been deposited in the European Genotype-phenotype Archive under the following accession: https://identifiers.org/ega.dataset:EGAD00001007025^60^ (study ID: EGAS00001005043). These data include NTHL1 sequencing using germline DNA, Alignment and variant calling, Whole-genome sequencing and targeted sequencing of tumor DNA, Genome-wide copy number analysis, Mutational signature analysis. In addition, the following data are not openly available to protect patient privacy: NTHL1 protein expression analysis, FCC patient database, cohorts summary, NTHL1 promoter methylation analysis. Data requests for these data should be made to the corresponding author.

## References

[1] Weren RD (2015). A germline homozygous mutation in the base-excision repair gene NTHL1 causes adenomatous polyposis and colorectal cancer. Nat. Genet..

[2] Fostira F (2018). Extending the clinical phenotype associated with biallelic NTHL1 germline mutations. Clin. Genet..

[3] Belhadj S (2019). NTHL1 biallelic mutations seldom cause colorectal cancer, serrated polyposis or a multi-tumor phenotype, in absence of colorectal adenomas. Sci. Rep..

[4] Grolleman JE (2019). Mutational signature analysis reveals NTHL1 deficiency to cause a multi-tumor phenotype. Cancer Cell.

[5] Groves A, Gleeson M, Spigelman AD (2019). NTHL1-associate polyposis: first Australian case report. Fam. Cancer.

[6] Drost J (2017). Use of CRISPR-modified human stem cell organoids to study the origin of mutational signatures in cancer. Science.

[7] Gallagher S (2020). Association of a polygenic risk score with breast cancer among women carriers of high- and moderate-risk breast cancer genes. JAMA Netw. Open.

[8] Mavaddat, N. et al. Prediction of breast cancer risk based on profiling with common genetic variants. *J. Natl Cancer Inst*. 10.1093/jnci/djv036 (2015). 10.1093/jnci/djv036 PMC4754625 25855707

[9] Muranen TA (2017). Genetic modifiers of CHEK2*1100delC-associated breast cancer risk. Genet. Med..

[10] Thompson D, Easton DF, Goldgar DE (2003). A full-likelihood method for the evaluation of causality of sequence variants from family data. Am. J. Hum. Genet..

[11] Pereira B (2016). The somatic mutation profiles of 2,433 breast cancers refines their genomic and transcriptomic landscapes. Nat. Commun..

[12] Li, N. et al. Combined tumor sequencing and case/control analyses of RAD51C in breast cancer. *J. Natl Cancer Inst*. 10.1093/jnci/djz045 (2019). 10.1093/jnci/djz045 PMC6910168 30949688

[13] Lee JEA (2018). Molecular analysis of PALB2-associated breast cancers. J. Pathol..

[14] Nik-Zainal S (2016). Landscape of somatic mutations in 560 breast cancer whole-genome sequences. Nature.

[15] Vollan HK (2015). A tumor DNA complex aberration index is an independent predictor of survival in breast and ovarian cancer. Mol. Oncol..

[16] Marquard AM (2015). Pan-cancer analysis of genomic scar signatures associated with homologous recombination deficiency suggests novel indications for existing cancer drugs. Biomark. Res..

[17] Telli ML (2016). Homologous recombination deficiency (HRD) score predicts response to platinum-containing neoadjuvant chemotherapy in patients with triple-negative breast cancer. Clin. Cancer Res..

[18] Goto M (2009). Altered expression of the human base excision repair gene NTH1 in gastric cancer. Carcinogenesis.

[19] Girard, E. et al. Familial breast cancer and DNA repair genes: Insights into known and novel susceptibility genes from the GENESIS study, and implications for multigene panel testing. *Int. J. Cancer*10.1002/ijc.31921 (2018). 10.1002/ijc.31921 PMC6587727 30303537

[20] Elsayed, F. A. et al. Monoallelic NTHL1 loss of function variants and risk of polyposis and colorectal cancer. *Gastroenterology*10.1053/j.gastro.2020.08.042 (2020). 10.1053/j.gastro.2020.08.042 PMC7899696 32860789

[21] Elledge SJ, Amon A (2002). The BRCA1 suppressor hypothesis: an explanation for the tissue-specific tumor development in BRCA1 patients. Cancer Cell.

[22] Polak P (2017). A mutational signature reveals alterations underlying deficient homologous recombination repair in breast cancer. Nat. Genet..

[23] Lee, J. E. A. et al. Molecular analysis of PALB2-associated breast cancers. *J. Pathol.*10.1002/path.5055 (2018). 10.1002/path.5055 29431189

[24] Mandelker D (2019). The landscape of somatic genetic alterations in breast cancers from CHEK2 germline mutation carriers. JNCI Cancer Spectr..

[25] Suspitsin EN (2014). Development of breast tumors in CHEK2, NBN/NBS1 and BLM mutation carriers does not commonly involve somatic inactivation of the wild-type allele. Med. Oncol..

[26] Payne SR, Kemp CJ (2005). Tumor suppressor genetics. Carcinogenesis.

[27] Willis A, Jung EJ, Wakefield T, Chen X (2004). Mutant p53 exerts a dominant negative effect by preventing wild-type p53 from binding to the promoter of its target genes. Oncogene.

[28] Goh AM, Coffill CR, Lane DP (2011). The role of mutant p53 in human cancer. J. Pathol..

[29] Papa A (2014). Cancer-associated PTEN mutants act in a dominant-negative manner to suppress PTEN protein function. Cell.

[30] Inoue K, Fry EA (2017). Haploinsufficient tumor suppressor genes. Adv. Med. Biol..

[31] Konishi H (2011). Mutation of a single allele of the cancer susceptibility gene BRCA1 leads to genomic instability in human breast epithelial cells. Proc. Natl Acad. Sci. USA.

[32] Sedic M, Kuperwasser C (2016). BRCA1-hapoinsufficiency: unraveling the molecular and cellular basis for tissue-specific cancer. Cell Cycle.

[33] Sedic M (2015). Haploinsufficiency for BRCA1 leads to cell-type-specific genomic instability and premature senescence. Nat. Commun..

[34] Jiang Z (2006). Expression analyses of 27 DNA repair genes in astrocytoma by TaqMan low-density array. Neurosci. Lett..

[35] Karger S (2012). Distinct pattern of oxidative DNA damage and DNA repair in follicular thyroid tumours. J. Mol. Endocrinol..

[36] Galick HA (2013). Germ-line variant of human NTH1 DNA glycosylase induces genomic instability and cellular transformation. Proc. Natl Acad. Sci. USA.

[37] Marsden CG (2020). Expression of a germline variant in the N-terminal domain of the human DNA glycosylase NTHL1 induces cellular transformation without impairing enzymatic function or substrate specificity. Oncotarget.

[38] Li N (2018). Mutations in RECQL are not associated with breast cancer risk in an Australian population. Nat. Genet..

[39] Kidd JR (2011). Analyses of a set of 128 ancestry informative single-nucleotide polymorphisms in a global set of 119 population samples. Investig. Genet..

[40] Kosoy R (2009). Ancestry informative marker sets for determining continental origin and admixture proportions in common populations in America. Hum. Mutat..

[41] Nassir R (2009). An ancestry informative marker set for determining continental origin: validation and extension using human genome diversity panels. BMC Genet..

[42] Li N (2018). Evaluating the breast cancer predisposition role of rare variants in genes associated with low-penetrance breast cancer risk SNPs. Breast Cancer Res..

[43] DePristo MA (2011). A framework for variation discovery and genotyping using next-generation DNA sequencing data. Nat. Genet..

[44] Van der Auwera GA (2013). From FastQ data to high confidence variant calls: the Genome Analysis Toolkit best practices pipeline. Curr. Protoc. Bioinform..

[45] Rimmer A (2014). Integrating mapping-, assembly- and haplotype-based approaches for calling variants in clinical sequencing applications. Nat. Genet..

[46] van Beers EH (2006). A multiplex PCR predictor for aCGH success of FFPE samples. Br. J. Cancer.

[47] Koboldt DC (2012). VarScan 2: somatic mutation and copy number alteration discovery in cancer by exome sequencing. Genome Res..

[48] Lek M (2016). Analysis of protein-coding genetic variation in 60,706 humans. Nature.

[49] Kuilman T (2015). CopywriteR: DNA copy number detection from off-target sequence data. Genome Biol..

[50] Burrell RA (2013). Replication stress links structural and numerical cancer chromosomal instability. Nature.

[51] Chin SF (2007). High-resolution aCGH and expression profiling identifies a novel genomic subtype of ER negative breast cancer. Genome Biol..

[52] Birkbak NJ (2012). Telomeric allelic imbalance indicates defective DNA repair and sensitivity to DNA-damaging agents. Cancer Discov..

[53] Abkevich V (2012). Patterns of genomic loss of heterozygosity predict homologous recombination repair defects in epithelial ovarian cancer. Br. J. Cancer.

[54] Popova T (2012). Ploidy and large-scale genomic instability consistently identify basal-like breast carcinomas with BRCA1/2 inactivation. Cancer Res..

[55] Rosenthal R, McGranahan N, Herrero J, Taylor BS, Swanton C (2016). DeconstructSigs: delineating mutational processes in single tumors distinguishes DNA repair deficiencies and patterns of carcinoma evolution. Genome Biol..

[56] R: A language and environment for statistical computing. (R Foundation for Statistical Computing, Vienna, Austria, 2016).

[57] Hansmann T (2012). Constitutive promoter methylation of BRCA1 and RAD51C in patients with familial ovarian cancer and early-onset sporadic breast cancer. Hum. Mol. Genet..

[58] Balduzzi S, Rucker G, Schwarzer G (2019). How to perform a meta-analysis with R: a practical tutorial. Evid. Based Ment. Health.

[59] Li, N. et al. Metadata record for the manuscript: Evaluation of the association of heterozygous germline variants in NTHL1 with breast cancer predisposition: an international multi-center study of 47,180 subjects. *figshare*. 10.6084/m9.figshare.14208293 (2021). 10.1038/s41523-021-00255-3 PMC8115524 33980861

[60] European Genotype-phenotype Archive. https://identifiers.org/ega.dataset:EGAD00001007025 (2021).

