# Supplementary material for: Evaluation of the association of heterozygous germline variants in NTHL1 with breast cancer predisposition: an international multi-center study of 47,180 subjects
Source: NPJ Breast Cancer. 2021 May 12;7:52. doi: 10.1038/s41523-021-00255-3 (PMC8115524; doi:10.1038/s41523-021-00255-3)
Supplement: Supplementary file 1 — Supplementary material [file 41523_2021_255_MOESM1_ESM.pdf]

Supplementary Tables

Supplementary Table 1. Personal and family history of *NTHL1* LoF variant carriers in BEACCON study.

Sample	Affected status	*NTHL1* variant	Diagnosis (age of onset) of cases or age of controls	No of breast cancer diagnosis in 1st-degree relatives
C35612	case	p.Ser22AlafsTer5	Breast(45, 60)	1
C31266	case	p.Gln90Ter	Ovary(53)	1
C20960	case	p.Gln90Ter	Breast(35)	1
C21261	case	p.Gln90Ter	Breast(56)	0
C30667	case	p.Gln90Ter	Breast(37)	0
C21552	case	p.Gln90Ter [1]	Breast(47, 53), Bowel(42, 55), Bladder(52), Uterus(53)	1
C21570	case	p.Gln90Ter	Breast(52)	1
C30165	case	p.Gln90Ter	Breast(54)	0
C21835	case	p.Gln90Ter	Breast(55), Ovary(54)	0
C31362	case	p.Gln90Ter	Breast(47)	1
C35407	case	p.Gln90Ter	Breast(38, 47)	0
C35456	case	p.Gln90Ter	Breast(68)	1
C36876	case	p.Gln90Ter	Breast(68), Skin, non-melanoma (age unknown)	2
C37500	case	p.Gln90Ter	Breast(30)	0
C37696	case	p.Gln90Ter	Breast(33)	0
C36922	case	p.Gln90Ter	Breast(45)	0
C31544	case	p.Gln90Ter	Peritoneal(62)	NA
C31956	case	p.Gln90Ter	Breast(24)	NA
C32018	case	p.Gln90Ter	Breast(57)	NA
C32110	case	p.Gln90Ter	Ovary(58)	NA
C32170	case	p.Gln90Ter	Breast(44)	NA
C32815	case	p.Gln90Ter	Breast(55)	NA
C32819	case	p.Gln90Ter	Breast(51)	NA

Sample	Affected status	*NTHL1* variant	Diagnosis (age of onset) of cases or age of controls	No of breast cancer diagnosis in 1st-degree relatives
C33461	case	p.Gln90Ter	Breast(39)	1
C34180	case	p.Gln90Ter	Breast(40)	1
C34833	case	p.Gln90Ter	Breast(31)	0
N36632	control	p.Gln90Ter	56	1
N30349	control	p.Gln90Ter	64	0
N34375	control	p.Gln90Ter	62	0
N34388	control	p.Gln90Ter	58	0
N35792	control	p.Gln90Ter	66	0
N30392	control	p.Gln90Ter	59	2
N37007	control	p.Gln90Ter	64	0
N33080	control	p.Gln90Ter	58	0
N35136	control	p.Gln90Ter	56	1
N33892	control	p.Gln90Ter	60	0
N37075	control	p.Gln90Ter	64	1
C30797	case	p.Arg129ThrfsTer42	Breast(31)	0
N33902	control	p.Tyr130Ter	81	0
N35337	control	p.Tyr130Ter	58	1
C33562	case	p.Arg153Ter	Breast(42)	NA
C34178	case	p.Arg153Ter	Breast(age unknown), Ovary(83)	0
C34086	case	p.Lys254Ter	Breast(51), Ovary(age unknown)	NA
C31264	case	p.Gln287Ter	Breast(51)	0
C21256	case	p.Gln287Ter	Breast(57)	1
C20188	case	p.Gln287Ter	Breast(51), Mouth(46, 59)	2
C21809	case	p.Gln287Ter	Breast(36)	1
C21298	case	p.Gln287Ter	Breast(28)	1
C37112	case	p.Gln287Ter	Breast(49)	1
C32774	case	p.Gln287Ter	Ovary(68)	1

Sample	Affected status	*NTHL1* variant	Diagnosis (age of onset) of cases or age of controls	No of breast cancer diagnosis in 1st-degree relatives
C33342	case	p.Gln287Ter	Breast(28)	NA
C33351	case	p.Gln287Ter	Breast(41)	0
N35940	control	p.Gln287Ter	69	0
N33964	control	p.Gln287Ter	50	0

[1] homozygous variant

Supplementary Table 2. Missense variants of *NTHL1* identified in cases and controls in BEACCON study.

CDS change [1]	Protein change [2]	Case	Control	Exon (of 6)	dbSNP ID	Condel	PolyPhen2	SIFT	CADD	REVEL	GnomAD [3]
c.5G>A	p.Cys2Tyr	1	0	1	.	Neu	Benign	Dele	13.98	0.03	0
c.31G>T	p.Ala11Ser	0	1	1	.	Neu	Benign	Toler	10.07	0.02	9.80E-06
c.53C>G	p.Thr18Ser	2	1	1	.	Neu	Benign	Dele	15.69	0.08	1.55E-04
c.55C>T	p.Arg19Trp	2	1	1	.	Dele	Prob Dam	Dele	17.03	0.20	3.03E-05
c.61C>T	p.Arg21Trp	3	2	1	rs3087469	Neu	Benign	Toler	13.62	0.12	5.68E-05
c.68T>C	p.Leu23Pro	2	2	1	.	Neu	Benign	Toler	13.04	0.10	1.93E-04
c.98G>A	p.Arg33Lys	1	0	1	rs2302172	Neu	Benign	Toler	9.73	0.04	4.44E-04
c.118C>T	p.Arg40Trp	1	0	1	.	Neu	Benign	Dele	13.90	0.05	0
c.125G>A	p.Arg42Lys	1	0	1	.	Neu	Benign	Toler	14.94	0.02	5.26E-06
c.257T>C	p.Val86Ala	1	0	2	.	Neu	Benign	Toler	9.86	0.01	0
c.276G>C	p.Trp92Cys	0	1	2	.	Dele	Prob Dam	Dele	19.03	0.54	1.86E-05
c.298C>T	p.Arg100Cys	19	14	2	rs148104494	Dele	Prob Dam	Dele	17.06	0.74	9.17E-04
c.368C>T	p.Ala123Val	0	1	2	.	Neu	Benign	Toler	16.47	0.09	2.96E-05
c.373C>T	p.Pro125Ser	0	1	2	rs149277519	Dele	Poss Dam	Toler	10.24	0.63	7.10E-05
c.469C>T	p.Arg157Trp	1	0	3	.	Neu	Benign	Toler	13.78	0.40	1.87E-05

CDS change [1]	Protein change [2]	Case	Control	Exon (of 6)	dbSNP ID	Condel	PolyPhen2	SIFT	CADD	REVEL	GnomAD [3]
c.470G>C	p.Arg157Pro	1	0	3	.	Dele	Poss Dam	Dele	16.82	0.60	7.64E-05
c.479C>T	p.Thr160Met	0	1	3	.	Dele	Prob Dam	Dele	21.10	0.79	4.49E-05
c.512C>T	p.Thr171Met	1	0	3	.	Neu	Benign	Toler	11.24	0.42	1.70E-05
c.517G>A	p.Gly173Ser	1	0	3	.	Dele	Prob Dam	Dele	22.90	0.67	0
c.527T>C	p.Ile176Thr	18	13	3	rs1805378	Dele	Prob Dam	Dele	18.50	0.88	1.81E-03
c.554A>G	p.Lys185Arg	2	0	4	.	Dele	Prob Dam	Dele	21.50	0.90	4.24E-06
c.601G>A	p.Gly201Ser	1	0	4	rs200007034	Neu	Benign	Toler	12.02	0.05	2.99E-05
c.607G>A	p.Asp203Asn	1	0	4	.	Dele	Prob Dam	Dele	24.40	0.27	1.87E-05
c.610A>C	p.Ile204Leu	1	0	4	.	Dele	Prob Dam	Dele	20.50	0.23	0
c.676A>G	p.Met226Val	3	1	4	.	Dele	Prob Dam	Dele	13.84	0.77	9.78E-05
c.737C>T	p.Ala246Val	1	0	5	.	Neu	Benign	Toler	13.28	0.52	4.22E-06
c.748A>G	p.Arg250Gly	1	0	5	.	Neu	Benign	Toler	0.01	0.31	0
c.770A>G	p.Lys257Arg	2	3	5	.	Neu	Benign	Toler	6.97	0.21	9.69E-05
c.772T>A	p.Ser258Thr	3	1	5	rs199698117	Neu	Benign	Toler	0.01	0.15	8.20E-05
c.787C>T	p.Arg263Cys	1	0	5	.	Dele	Prob Dam	Dele	12.97	0.52	5.59E-05
c.828C>G	p.His276Gln	0	2	6	.	Neu	Benign	Toler	0.01	0.29	0
c.847G>A	p.Val283Met	1	0	6	.	Dele	Prob Dam	Dele	16.85	0.80	4.27E-06

CDS change [1]	Protein change [2]	Case	Control	Exon (of 6)	dbSNP ID	Condel	PolyPhen2	SIFT	CADD	REVEL	GnomAD [3]
c.856G>A	p.Gly286Ser	1	0	6	rs139309757	Dele	Prob Dam	Dele	24.20	0.97	3.77E-05
c.878T>C	p.Val293Ala	0	1	6	.	Neu	Benign	Dele	11.80	0.31	0
c.886C>T	p.Arg296Cys	1	0	6	.	Dele	Poss Dam	Dele	18.52	0.79	0
c.895G>A	p.Ala299Thr	1	0	6	.	Neu	Benign	Toler	9.37	0.29	0
c.935T>C	p.Leu312Pro	1	1	6	.	Dele	Poss Dam	Dele	14.05	0.05	0

[1] ENST00000219066.1(NM_002528.5); Neu, neutral; Dele, deleterious; Prob Dam, probably damaging; Poss Dam, possibly damaging; Toler, tolerated;

[2] ENSP00000219066.1(NP_002519.1);

[3] GnomAD, minor allele frequency in non-cancer cohorts in GnomAD database. One case carrier harbored two variants: p.Arg19Trp and p.Arg100Cys.

Supplementary Table 3. Overall and likely pathogenic missense variants of *NTHL1* filtered by population frequency and *in silico* prediction tools in cases and controls in BEACCON study

NTHL1 missense variants	Case n = 4985		Control n = 4786		OR	CI	P
	Carriers	%	Carriers	%			
Overall unselected [1]	75	1.50	47	0.98	1.54	1.05-2.27	0.02
Rare (MAF ≤ 0.001) [2]	39	0.78	20	0.42	1.88	1.07-3.41	0.03
Condel (deleterious)	52	1.04	33	0.69	1.52	0.96-2.43	0.06
PolyPhen2 (damaging)	52	1.04	31	0.65	1.62	1.02-2.62	0.04
SIFT (deleterious)	56	1.12	34	0.71	1.59	1.02-2.51	0.03
CADD (≥10)	66	1.32	41	0.86	1.55	1.03-2.36	0.03
REVEL (≥0.75)	26	0.52	15	0.31	1.67	0.85-3.39	0.12

[1] Includes all the missense variants identified in *NTHL1* without any population frequency filters or *in silico* prediction filters.

[2] MAF, minor allele frequency in gnomAD database.

Supplementary Table 4. Histo-pathological subtypes and Hormone receptor status of the breast cancers derived from heterozygous and homozygous LoF *NTHL1* variant carriers.

Het, heterozygous variant; Null, homozygous variant; IDC, invasive ductal carcinoma; ILC, invasive lobular carcinoma; ER, oestrogen receptor; PR, progesterone receptor; HER2, human epidermal growth factor receptor 2. NA, not available.

Sample	*NTHL1* status	Age of diagnosis	Laterality	Histological subtype	Grade	ER	PR	HER2
C20960	Het	35	Right	IDC	G3	ER+	PR+	HER2-
C21256	Het	57	Right	IDC	G2	ER+	NA	HER2-
C21261	Het	56	Right	IDC	G3	ER+	PR+	HER2-
C20188	Het	51	Right	IDC	G3	ER-	PR-	HER2-
C30667	Het	37	Left	IDC	G2	ER-	PR+	HER2+
C21809	Het	36	Left	IDC	G2	ER+	PR+	HER2+
C21298	Het	28	Right	IDC (Medullary)	NA	ER-	PR+	NA
C30165	Het	54	Left	IDC	G3	ER-	PR-	HER2-

C21835	Het	55	NA	IDC	G2	ER+	PR+	HER2-
C30797	Het	31	Right	IDC	G2	ER+	PR+	HER2+
C37112	Het	49	Right	IDC	G2	ER+	PR+	HER2-
C31362	Het	47	Right	IDC	G2	ER+	PR+	HER2-
C35407	Het	38	Left	IDC	G1	ER+	PR+	HER2-
C35407	Het	47	NA	IDC	G3	ER-	PR-	HER2-
C35456	Het	68	Right	IDC	G2	ER+	PR+	HER2-
C35612	Het	45	Right	IDC	G3	ER-	PR+	NA
C35612	Het	60	Left	IDC	G3	ER-	PR-	HER2-
C36876	Het	68	Right	IDC (Mucinous)	G2	ER+	PR+	HER2-
C37500	Het	30	Left	IDC	G2	ER+	PR+	HER2-
C37696	Het	33	Left	IDC	G2	ER+	PR+	HER2-
C36922	Het	45	Right	IDC (Medullary)	G3	ER-	PR-	HER2-
C21552	Null	47	Left	ILC	G2	ER+	PR+	HER2-
C21552	Null	53	Right	IDC	G2	ER+	PR+	HER2-

Supplementary Table 5. Subjects and cohorts ascertain standard, sequencing method and coverage information for the ten multi-center international studies.

Study	Case ascertain standard [1]	Country of origin	N. of cases	N. of controls	Sequencing method	Case 10x coverage (Depth)	Control 10x coverage (Depth)
BEACCON, hereditary BrEAst Case CONtrol study	Hereditary	Australia	4,985	4,786	Targeted sequencing (HaloPlex HS Targeted Enrichment Assay, Agilent Technologies)	99.32% (396.13)	99.19% (358.64)
GC-HBOC, German Consortium for Hereditary Breast and Ovarian Cancer	Hereditary	Germany	3,199	2,767	Targeted sequencing (Agilent SureSelect QXT target Enrichment assay (Agilent Technologies)	98,85% (193.08)	98,88% (205,24)
GENESIS, French familial BRCAx	Hereditary	France	1,207	1,199	Targeted sequencing (SureSelect target Enrichment system, Agilent Technologies [2])	99.6% (223.21)	99.6% (224,98)
DFBBCS, the Dutch Familial Bilateral Breast Cancer Study	Hereditary	Netherland	1,012	962	Targeted sequencing (Agilent SureSelect QXT target Enrichment assay (Agilent Technologies)	98,78% (382,31)	98,82% (348,30)
VHIO, familial breast cancer and control study of the Vall d'Hebron Institute of Oncology of Barcelona	Hereditary	Spain	1,012	488	Targeted sequencing (Hi-Plex [3])	94.44% (1186)	92.86% (1097)
OFBCR, Ontario Familial Breast Cancer Registry	Hereditary	Canada,Ontario	600	592	Targeted sequencing (Agilent SureSelect QXT target Enrichment assay (Agilent Technologies)	98,67% (355,76)	98,74% (294,88)

SEARCH, UK Population-based Breast Cancer Study	Population-based	United Kingdom	12,523	6,474	Targeted sequencing (Fluidigm Juno Access Array)	94.51% (171.41)	95.01% (152.21)
ABCFR, Australian Breast Cancer Family Registry	Population-based cases	Australia	1,421	833	Targeted sequencing (HaloPlex HS Targeted Enrichment Assay, Agilent Technologies)<br>Targeted sequencing (Hi-Plex [3])	99.73% (474)	98.47% (2470)
HABC, Hispanic American Breast Cancer study	Mostly Hereditary	USA(Hispanic American)	1,045	1,189	Whole exome sequencing	NA	NA
CARTaGENE, Québec Population-based Breast Cancer Study	Population-based	Canada, Québec (French-Canadian)	451	469	Targeted sequencing (Agilent SureSelect QXT target Enrichment assay (Agilent Technologies)	98,35% (312,90)	98,57% (287,32)

[1] case ascertain standards included hereditary, the case subjects in the study were predominantly ascertained in different standard to enrich for high-risk breast cancer patients and population-based, the case subjects were recruited in relevant population without consideration of enrichment for family history.

[2] Girard, E. et al. Familial breast cancer and DNA repair genes: Insights into known and novel susceptibility genes from the GENESIS study, and implications for multigene panel testing. Int J Cancer, doi:10.1002/ijc.31921 (2018).

[3] Hammet, F. et al. Hi-Plex2: a simple and robust approach to targeted sequencing-based genetic screening. Biotechniques 67, 118-122, doi:10.2144/btn-2019-0026 (2019).

Supplementary Table 6. All LoF and missense variants identified in 47,180 case and control subjects in ten international case-control cohorts

CDS change [1]	Protein change [2]	dbSNP IDs	Consequence	Total		BEACCON [3]		GC-HBOC		GENESIS		DFBBCS		VHIO		OFBCR		SEARCH		ABCFR		HABC		CARTaGENE		gnomAD	gnomAD (NFE)
				Case	Control	Case	Control	Case	Control	Case	Control	Case	Control	Case	Control	Case	Control	Case	Control	Case	Control	Case	Control	Case	Control		
c.64_83del	p.Ser22AlafsTer5	-	Frameshift(LoF)	1	0	1	0	0	0	0	0	0	0	0	0	0	0	0	0	0	0	0	0	0	0	0	0
c.140-1G>A	-	-	Essential splice site(LoF)	2	0	0	0	1	0	0	0	1	0	0	0	0	0	0	0	0	0	0	0	0	0	0	0
c.227del	p.Gly76ValfsTer27	-	Frameshift(LoF)	0	1	0	0	0	0	0	1	0	0	0	0	0	0	0	0	0	0	0	0	0	0	0	0
c.235dup	p.Ala79GlyfsTer2	rs745671590	Stop Gained(LoF)	1	0	0	0	1	0	0	0	0	0	0	0	0	0	0	0	0	0	0	0	0	0	4.22E-06	9.73E-06
c.235_236del	p.Ala79Ter	-	Frameshift(LoF)	0	0	0	0	0	0	0	0	0	0	0	0	0	0	0	0	0	0	0	0	0	0	0	0
c.250del	p.Val84CysfsTer19	-	Frameshift(LoF)	2	0	0	0	0	0	0	0	0	0	0	0	0	0	2	0	0	0	0	0	0	0	0	0
c.268C>T	p.Gln90Ter	rs150766139	Stop Gained(LoF)	102	81	25	11	9	17	3	4	4	3	2	2	2	4	50	34	2	2	3	3	2	1	1.44E-03	1.99E-03
c.380_383dup	p.Arg129ThrfsTer42	rs759955745	Frameshift(LoF)	1	0	1	0	0	0	0	0	0	0	0	0	0	0	0	0	0	0	0	0	0	0	4.48E-06	1.04E-05
c.390C>A	p.Tyr130Ter	rs371328106	Stop Gained(LoF)	2	2	0	1	0	0	0	0	0	0	0	0	0	0	2	1	0	0	0	0	0	0	2.36E-05	5.38E-05
c.390C>G	p.Tyr130Ter	-	Stop Gained(LoF)	0	1	0	1	0	0	0	0	0	0	0	0	0	0	0	0	0	0	0	0	0	0	0	0
c.433C>T	p.Gln145Ter	rs758667255	Stop Gained(LoF)	3	1	0	0	0	0	0	0	0	0	0	0	0	0	2	1	1	0	0	0	0	0	4.25E-06	9.80E-06
c.457C>T	p.Arg153Ter	rs374489979	Stop Gained(LoF)	2	0	2	0	0	0	0	0	0	0	0	0	0	0	0	0	0	0	0	0	0	0	1.28E-05	9.89E-06
c.469_470del	p.Arg157GlyfsTer12	-	Frameshift(LoF)	1	0	0	0	0	0	0	0	0	0	1	0	0	0	0	0	0	0	0	0	0	0	0	0
c.550-1G>A	-	rs779757251	Essential splice site(LoF)	2	1	0	0	0	0	1	0	0	0	0	0	0	0	0	0	0	0	0	0	1	1	8.07E-05	6.85E-05
c.566del	p.Ile189ThrfsTer61	-	Frameshift(LoF)	2	0	0	0	2	0	0	0	0	0	0	0	0	0	0	0	0	0	0	0	0	0	0	0
c.628G>T	p.Glu210Ter	-	Stop Gained(LoF)	0	1	0	0	0	0	0	0	0	0	0	0	0	0	0	1	0	0	0	0	0	0	3.19E-05	6.49E-05
c.697del	p.Val233CysfsTer17	-	Frameshift(LoF)	0	1	0	0	0	0	0	0	0	0	0	1	0	0	0	0	0	0	0	0	0	0	0	0
c.760A>T	p.Lys254Ter	-	Stop Gained(LoF)	1	0	1	0	0	0	0	0	0	0	0	0	0	0	0	0	0	0	0	0	0	0	0	0

c.806G>A	p.Trp269Ter	rs753029097	Stop Gained(LoF)	2	0	0	0	0	0	0	0	0	0	0	0	2	0	0	0	0	0	0	0	0	0	2.11E-05	1.95E-05
c.859C>T	p.Gln287Ter	rs146347092	Stop Gained(LoF)	14	4	9	2	0	0	0	0	0	0	0	0	0	0	3	2	2	0	0	0	0	0	1.62E-04	1.88E-04
c.902del	p.Leu301ProfsTer40	-	Frameshift(LoF)	1	0	0	0	1	0	0	0	0	0	0	0	0	0	0	0	0	0	0	0	0	0	0	0
c.5G>A	p.Cys2Tyr	-	Missense	1	0	1	0	0	0	0	0	0	0	0	0	0	0	0	0	0	0	0	0	0	0	0	0
c.8G>C	p.Ser3Thr	rs780026159	Missense	2	1	0	0	0	0	1	1	0	0	1	0	0	0	0	0	0	0	0	0	0	0	8.48E-06	1.92E-05
c.31G>T	p.Ala11Ser	rs753350404	Missense	1	2	0	1	0	0	1	1	0	0	0	0	0	0	0	0	0	0	0	0	0	0	9.80E-06	1.14E-05
c.31G>A	p.Ala11Thr	rs753350404	Missense	1	0	0	0	0	0	0	0	0	0	1	0	0	0	0	0	0	0	0	0	0	0	0	0
c.53C>G	p.Thr18Ser	rs370539291	Missense	2	1	2	1	0	0	0	0	0	0	0	0	0	0	0	0	0	0	0	0	0	0	1.55E-04	0
c.55C>T	p.Arg19Trp	rs549760347	Missense	4	1	2	1	0	0	1	0	0	0	0	0	0	0	0	0	1	0	0	0	0	0	3.03E-05	1.98E-05
c.61C>T	p.Arg21Trp	rs3087469	Missense	3	3	3	2	0	0	0	0	0	0	0	0	0	0	0	0	0	1	0	0	0	0	5.68E-05	1.30E-04
c.68T>C	p.Leu23Pro	rs781328762	Missense	3	3	2	2	0	0	1	1	0	0	0	0	0	0	0	0	0	0	0	0	0	0	1.93E-04	2.12E-04
c.92G>A	p.Gly31Glu	-	Missense	0	1	0	0	0	0	0	1	0	0	0	0	0	0	0	0	0	0	0	0	0	0	0	0
c.98G>A	p.Arg33Lys	rs2302172	Missense	2	0	1	0	0	0	0	0	0	0	1	0	0	0	0	0	0	0	0	0	0	0	4.44E-04	5.17E-05
c.118C>T	p.Arg40Trp	-	Missense	1	0	1	0	0	0	0	0	0	0	0	0	0	0	0	0	0	0	0	0	0	0	0	0
c.125G>A	p.Arg42Lys	-	Missense	1	0	1	0	0	0	0	0	0	0	0	0	0	0	0	0	0	0	0	0	0	0	5.26E-06	1.24E-05
c.143C>T	p.Ala48Val	rs375615004	Missense	0	1	0	0	0	0	0	0	0	0	0	0	0	0	0	0	0	0	0	1	0	0	3.41E-05	9.81E-06
c.169C>T	p.Arg57Cys	rs371105614	Missense	1	2	0	0	0	0	0	0	0	0	0	0	0	0	1	2	0	0	0	0	0	0	7.48E-06	0
c.173C>T	p.Pro58Leu	rs139165943	Missense	2	1	0	0	0	0	1	0	0	0	0	0	0	0	1	0	0	0	0	1	0	0	1.23E-04	0
c.175C>T	p.Arg59Trp	rs376907606	Missense	1	1	0	0	0	0	1	1	0	0	0	0	0	0	0	0	0	0	0	0	0	0	2.12E-05	3.90E-05
c.176G>A	p.Arg59Gln	rs773364076	Missense	0	1	0	0	0	0	0	0	0	0	0	0	0	0	0	0	0	0	0	1	0	0	1.69E-05	9.76E-06
c.193C>T	p.Arg65Cys	rs767591879	Missense	3	0	0	0	0	0	0	0	0	0	0	0	0	0	0	0	0	0	3	0	0	0	7.47E-05	8.47E-06
c.202T>C	p.Tyr68His	-	Missense	1	0	0	0	0	0	0	0	0	0	0	0	0	0	1	0	0	0	0	0	0	0	0	0
c.208G>A	p.Gly70Ser	rs368897948	Missense	0	1	0	0	0	0	0	1	0	0	0	0	0	0	0	0	0	0	0	0	0	0	2.54E-05	2.92E-05
c.212C>T	p.Ser71Leu	rs746458904	Missense	2	1	0	0	0	0	0	0	0	0	0	0	0	0	2	1	0	0	0	0	0	0	4.10E-05	6.77E-05
c.229_230delinsTT	p.Glu77Leu	-	Missense	1	0	0	0	0	0	0	0	0	0	0	0	0	0	1	0	0	0	0	0	0	0	0	0
c.257T>C	p.Val86Ala	-	Missense	1	0	1	0	0	0	0	0	0	0	0	0	0	0	0	0	0	0	0	0	0	0	0	0
c.266C>T	p.Pro89Leu	-	Missense	2	0	0	0	0	0	0	0	0	0	0	0	0	0	2	0	0	0	0	0	0	0	4.22E-06	9.73E-06

c.276G>C	p.Trp92Cys	rs372698989	Missense	0	1	0	1	0	0	0	0	0	0	0	0	0	0	0	0	0	0	0	0	0	0	1.86E-05	2.54E-05
c.292A>G	p.Asn98Asp	rs141903513	Missense	0	1	0	0	0	0	0	1	0	0	0	0	0	0	0	0	0	0	0	0	0	0	2.24E-05	0
c.296T>A	p.Ile99Asn	-	Missense	1	0	0	0	0	0	0	0	0	0	0	0	0	0	1	0	0	0	0	0	0	0	0	0
c.298C>T	p.Arg100Cys	rs148104494	Missense	82	48	19	14	8	7	3	3	2	2	0	0	2	1	44	19	2	0	2	2	0	0	9.17E-04	1.31E-03
c.299G>A	p.Arg100His	rs145644817	Missense	1	1	0	0	1	1	0	0	0	0	0	0	0	0	0	0	0	0	0	0	0	0	5.96E-05	1.69E-05
c.306G>C	p.Met102Ile	-	Missense	1	0	0	0	1	0	0	0	0	0	0	0	0	0	0	0	0	0	0	0	0	0	4.22E-06	9.73E-06
c.332A>G	p.Asp111Gly	rs747659604	Missense	0	1	0	0	0	0	0	0	0	0	0	0	0	0	0	0	0	0	0	1	0	0	4.22E-06	0
c.356A>G	p.Tyr119Cys	rs138812334	Missense	0	2	0	0	0	0	0	0	0	0	0	0	0	0	0	2	0	0	0	0	0	0	4.22E-05	6.82E-05
c.368C>T	p.Ala123Val	rs745986873	Missense	0	1	0	1	0	0	0	0	0	0	0	0	0	0	0	0	0	0	0	0	0	0	2.96E-05	6.84E-05
c.370C>A	p.Pro124Thr	rs143696592	Missense	3	1	0	0	0	0	0	1	0	0	0	0	0	0	3	0	0	0	0	0	0	0	2.12E-05	3.91E-05
c.373C>T	p.Pro125Ser	rs149277519	Missense	5	4	0	1	0	0	1	1	0	0	0	0	0	0	4	2	0	0	0	0	0	0	7.10E-05	1.44E-04
c.374C>A	p.Pro125Gln	rs777263711	Missense	0	2	0	0	0	0	0	2	0	0	0	0	0	0	0	0	0	0	0	0	0	0	8.47E-06	0
c.379G>A	p.Val127Ile	-	Missense	0	1	0	0	0	0	0	0	0	0	0	0	0	0	0	1	0	0	0	0	0	0	0	0
c.383G>A	p.Arg128His	-	Missense	1	0	0	0	0	0	0	0	0	0	0	0	0	0	1	0	0	0	0	0	0	0	0	0
c.385A>G	p.Arg129Gly	-	Missense	0	1	0	0	0	0	0	0	0	0	0	0	0	0	0	1	0	0	0	0	0	0	0	0
c.401T>A	p.Leu134Gln	-	Missense	1	0	0	0	1	0	0	0	0	0	0	0	0	0	0	0	0	0	0	0	0	0	4.32E-06	9.97E-06
c.401T>G	p.Leu134Arg	-	Missense	1	0	0	0	0	0	0	0	0	0	0	0	0	0	1	0	0	0	0	0	0	0	0	0
c.416C>T	p.Ser139Phe	-	Missense	1	0	0	0	1	0	0	0	0	0	0	0	0	0	0	0	0	0	0	0	0	0	0	0
c.435G>C	p.Gln145His	-	Missense	0	2	0	0	0	0	0	0	0	0	0	0	0	0	0	2	0	0	0	0	0	0	0	0
c.440C>T	p.Thr147Met	rs756597137	Missense	1	1	0	0	0	1	0	0	0	0	0	0	0	0	1	0	0	0	0	0	0	0	6.00E-05	6.82E-05
c.443C>T	p.Ala148Val	rs750992242	Missense	0	1	0	0	0	0	0	0	0	1	0	0	0	0	0	0	0	0	0	0	0	0	1.28E-05	2.94E-05
c.448G>A	p.Ala150Thr	rs764907191	Missense	0	1	0	0	0	0	0	0	0	0	0	0	0	0	0	0	0	0	0	1	0	0	1.29E-05	0
c.455A>G	p.Gln152Arg	-	Missense	0	1	0	0	0	0	0	0	0	0	0	0	0	0	0	1	0	0	0	0	0	0	4.28E-06	9.88E-06
c.467C>T	p.Ala156Val	rs748576083	Missense	1	0	0	0	0	0	0	0	0	0	0	0	0	0	1	0	0	0	0	0	0	0	1.70E-05	3.92E-05
c.469C>T	p.Arg157Trp	rs376048896	Missense	2	0	1	0	0	0	0	0	0	0	0	0	0	0	1	0	0	0	0	0	0	0	1.87E-05	3.40E-05
c.470G>C	p.Arg157Pro	rs150437839	Missense	1	0	1	0	0	0	0	0	0	0	0	0	0	0	0	0	0	0	0	0	0	0	7.64E-05	0
c.479C>T	p.Thr160Met	rs756403102	Missense	1	2	0	1	0	0	0	0	0	0	0	0	0	0	1	1	0	0	0	0	0	0	4.49E-05	6.79E-05

c.497A>G	p.Gln166Arg	-	Missense	1	0	0	0	0	0	0	0	0	0	0	0	0	0	1	0	0	0	0	0	0	0	0	0
c.500C>T	p.Thr167Ile	rs968060928	Missense	1	0	0	0	0	0	0	0	0	0	0	0	0	0	1	0	0	0	0	0	0	0	6.37E-05	0
c.512C>T	p.Thr171Met	rs766136810	Missense	1	0	1	0	0	0	0	0	0	0	0	0	0	0	0	0	0	0	0	0	0	0	1.70E-05	0
c.517G>A	p.Gly173Ser	-	Missense	1	0	1	0	0	0	0	0	0	0	0	0	0	0	0	0	0	0	0	0	0	0	0	0
c.524T>C	p.Leu175Pro	-	Missense	0	1	0	0	0	0	0	0	0	0	0	0	0	0	0	1	0	0	0	0	0	0	0	0
c.527T>C	p.Ile176Thr	rs1805378	Missense	114	61	18	13	19	8	10	4	6	2	8	6	3	3	44	17	0	0	5	7	1	1	1.81E-03	2.22E-03
c.535G>C	p.Val179Leu	rs561923127	Missense	1	0	0	0	0	0	0	0	0	0	0	0	0	0	1	0	0	0	0	0	0	0	8.62E-06	1.96E-05
c.535G>A	p.Val179Ile	rs561923127	Missense	2	0	0	0	0	0	0	0	0	0	0	0	0	0	2	0	0	0	0	0	0	0	3.45E-05	2.94E-05
c.554A>G	p.Lys185Arg	-	Missense	3	0	2	0	0	0	0	0	0	0	0	0	0	0	0	0	1	0	0	0	0	0	4.24E-06	0
c.580G>A	p.Ala194Thr	rs554393986	Missense	1	0	0	0	0	0	0	0	0	0	0	0	0	0	1	0	0	0	0	0	0	0	1.06E-04	3.90E-05
c.595C>T	p.His199Tyr	-	Missense	0	0	0	0	0	0	0	0	0	0	0	0	0	0	0	0	0	0	0	0	0	0	4.23E-06	0
c.596A>G	p.His199Arg	-	Missense	0	1	0	0	0	0	0	0	0	0	0	0	0	0	0	1	0	0	0	0	0	0	4.23E-06	0
c.601G>A	p.Gly201Ser	rs200007034	Missense	2	0	1	0	0	0	0	0	0	0	0	0	0	0	1	0	0	0	0	0	0	0	2.99E-05	2.54E-05
c.607G>A	p.Asp203Asn	rs765358651	Missense	1	1	1	0	0	0	0	0	0	0	0	1	0	0	0	0	0	0	0	0	0	0	1.69E-05	2.92E-05
c.610A>C	p.Ile204Leu	-	Missense	1	0	1	0	0	0	0	0	0	0	0	0	0	0	0	0	0	0	0	0	0	0	0	0
c.646G>C	p.Gly216Arg	-	Missense	1	0	0	0	0	0	0	0	0	0	0	0	0	0	1	0	0	0	0	0	0	0	0	0
c.661A>G	p.Met221Val	rs756963327	Missense	2	0	0	0	2	0	0	0	0	0	0	0	0	0	0	0	0	0	0	0	0	0	8.51E-06	1.95E-05
c.676A>G	p.Met226Val	rs367577861	Missense	5	2	3	1	1	0	0	0	0	0	0	1	0	0	0	0	1	0	0	0	0	0	9.78E-05	0
c.704G>A	p.Gly235Asp	rs1055874267	Missense	1	0	0	0	0	0	1	0	0	0	0	0	0	0	0	0	0	0	0	0	0	0	4.30E-06	0
c.719C>T	p.Thr240Met	-	Missense	1	0	0	0	1	0	0	0	0	0	0	0	0	0	0	0	0	0	0	0	0	0	4.23E-06	0
c.737C>T	p.Ala246Val	-	Missense	1	0	1	0	0	0	0	0	0	0	0	0	0	0	0	0	0	0	0	0	0	0	4.22E-06	0
c.748A>G	p.Arg250Gly	-	Missense	1	0	1	0	0	0	0	0	0	0	0	0	0	0	0	0	0	0	0	0	0	0	0	0
c.770A>G	p.Lys257Arg	rs373067940	Missense	10	8	2	3	0	0	0	0	0	0	0	0	0	0	8	5	0	0	0	0	0	0	9.69E-05	2.20E-04
c.772T>A	p.Ser258Thr	rs199698117	Missense	5	2	3	1	0	0	1	0	0	0	0	0	0	0	1	1	0	0	0	0	0	0	8.20E-05	1.78E-04
c.787C>T	p.Arg263Cys	rs779992803	Missense	3	1	1	0	0	0	1	0	0	0	0	0	0	0	1	1	0	0	0	0	0	0	5.59E-05	1.10E-04
c.788G>A	p.Arg263His	rs756036462	Missense	1	0	0	0	0	0	0	0	0	0	0	0	0	0	1	0	0	0	0	0	0	0	3.38E-05	1.95E-05
c.793G>A	p.Ala265Thr	rs148474733	Missense	3	2	0	0	0	0	0	0	0	0	0	0	0	0	0	0	0	0	3	2	0	0	1.90E-04	0

c.828C>G	p.His276Gln	-	Missense	0	2	0	2	0	0	0	0	0	0	0	0	0	0	0	0	0	0	0	0	0	0	0	0
c.830A>T	p.Glu277Val	rs369032190	Missense	1	0	0	0	0	0	0	0	0	0	0	0	0	0	0	0	1	0	0	0	0	0	4.27E-06	9.90E-06
c.836A>G	p.Asn279Ser	rs149287105	Missense	1	0	0	0	0	0	0	0	0	0	0	0	0	0	1	0	0	0	0	0	0	0	8.53E-06	0
c.841C>T	p.Leu281Phe	rs939350999	Missense	1	0	0	0	1	0	0	0	0	0	0	0	0	0	0	0	0	0	0	0	0	0	0	0
c.847G>A	p.Val283Met	rs759411504	Missense	2	0	1	0	0	0	0	0	0	0	0	0	0	0	1	0	0	0	0	0	0	0	4.27E-06	9.87E-06
c.856G>A	p.Gly286Ser	rs139309757	Missense	5	1	1	0	2	1	0	0	0	0	0	0	0	0	2	0	0	0	0	0	0	0	3.77E-05	4.28E-05
c.878T>C	p.Val293Ala	-	Missense	0	1	0	1	0	0	0	0	0	0	0	0	0	0	0	0	0	0	0	0	0	0	0	0
c.886C>T	p.Arg296Cys	rs571813695	Missense	1	1	1	0	0	0	0	0	0	0	0	0	0	0	0	1	0	0	0	0	0	0	0	0
c.895G>A	p.Ala299Thr	rs763568491	Missense	1	0	1	0	0	0	0	0	0	0	0	0	0	0	0	0	0	0	0	0	0	0	0	0
c.901C>A	p.Leu301Ile	-	Missense	1	0	0	0	1	0	0	0	0	0	0	0	0	0	0	0	0	0	0	0	0	0	0	0
c.906C>G	p.Asn302Lys	rs759356343	Missense	0	1	0	0	0	0	0	0	0	0	0	0	0	0	0	1	0	0	0	0	0	0	4.34E-06	9.82E-06
c.920C>T	p.Pro307Leu	rs367629024	Missense	1	0	0	0	0	0	0	0	0	0	1	0	0	0	0	0	0	0	0	0	0	0	7.68E-06	1.71E-05
c.925G>A	p.Ala309Thr	-	Missense	1	1	0	0	0	1	0	0	0	0	0	0	0	0	1	0	0	0	0	0	0	0	4.38E-06	0
c.935T>C	p.Leu312Pro	-	Missense	1	1	1	1	0	0	0	0	0	0	0	0	0	0	0	0	0	0	0	0	0	0	0	0

[1] ENSP00000219066.1 (NM_002528.5)

[2] ENST00000219066.1 (NP_002519.1)

[3] One case had a homozygous p.Gln90Ter variant and one case harbored two missense variants (p.Arg19Trp and p.Arg100Cys) in BEACCON study.

Supplementary Table 7. List of all primers used in *NTHL1* variants validation and promoter methylation analysis.

Primer ID	Sequence	Experiment
NTHL1_ex1	CCGGGATGTGTAGTCCGC(Forward)	Variants validation
	CTATCCCGCCTCCTCCCA(Reverse)	
NTHL1_ex3A	CTCAAGTGTGGCCCAGGG(Forward)	Variants validation
	GGCTCACCCTCCAGAAACC(Reverse)	
NTHL1_ex5	GGAGTGTGCCCCTGTTCA(Forward)	Variants validation
	GCTCTTCTCCCTAGGAAGCC(Reverse)	
NTHL1_ex4	AGCTTGCACACGTCCTCA(Forward)	Variants validation
	GAATCCCAAGAGCAGCCAGT(Reverse)	
NTHL1_ex6	GTGGCTGCCTAGGTATGAGT(Forward)	Variants validation
	AAAGCCACTTCACAGACGGT(Reverse)	
NTHL1_ex2	CACCCCTACCCTACCTTCAC(Forward)	Variants validation
	GGCACTGGAGTCATAGCAGT(Reverse)	
NTHL1_ex3	CACAAGCAGGTACGCAGGTA(Forward)	Variants validation
	CCTGAGATGCTTGACCCTCA(Reverse)	
NTHL1_methy	GGAAAGTTYGGGAGATATTAGGTTTGGTTTAGAGAG(Forward)	Promoter methylation
	AAACTCCRAACATCCCTTAATTTTAAATCATAAC(Reverse)	

Supplementary Figures

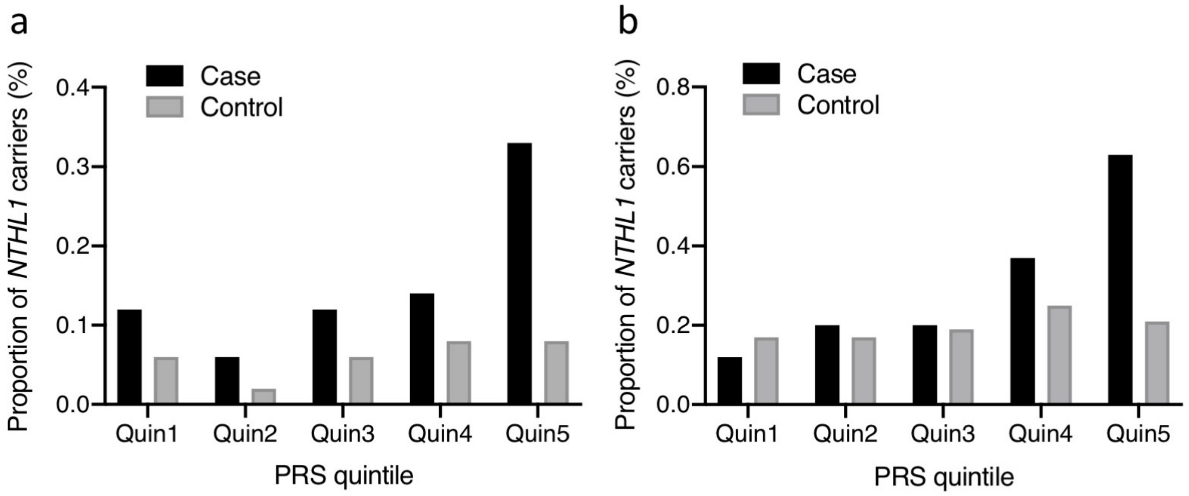

Supplementary Figure 1. The distribution of the cases and controls carrying a *NTHL1* LoF variant (a) or a *NTHL1* missense variant (b) in PRS quintiles (according to all controls in the BEACCON study).

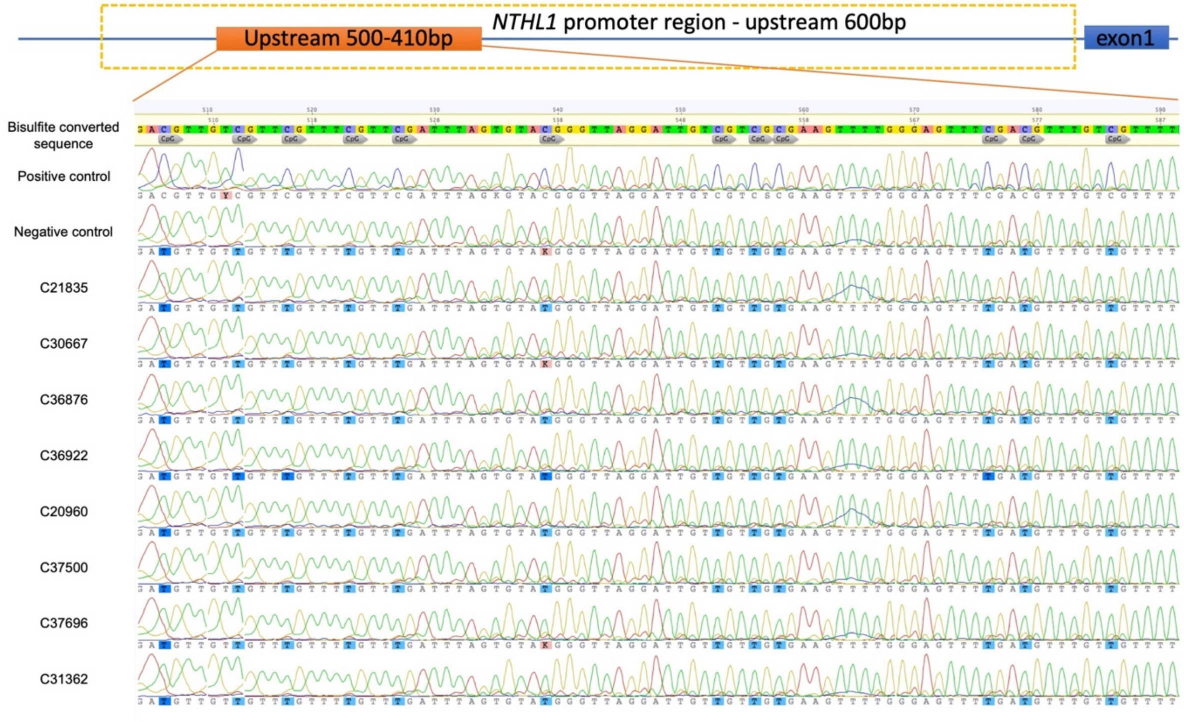

Supplementary Figure 2. ***NTHL1* promoter methylation sequencing**.

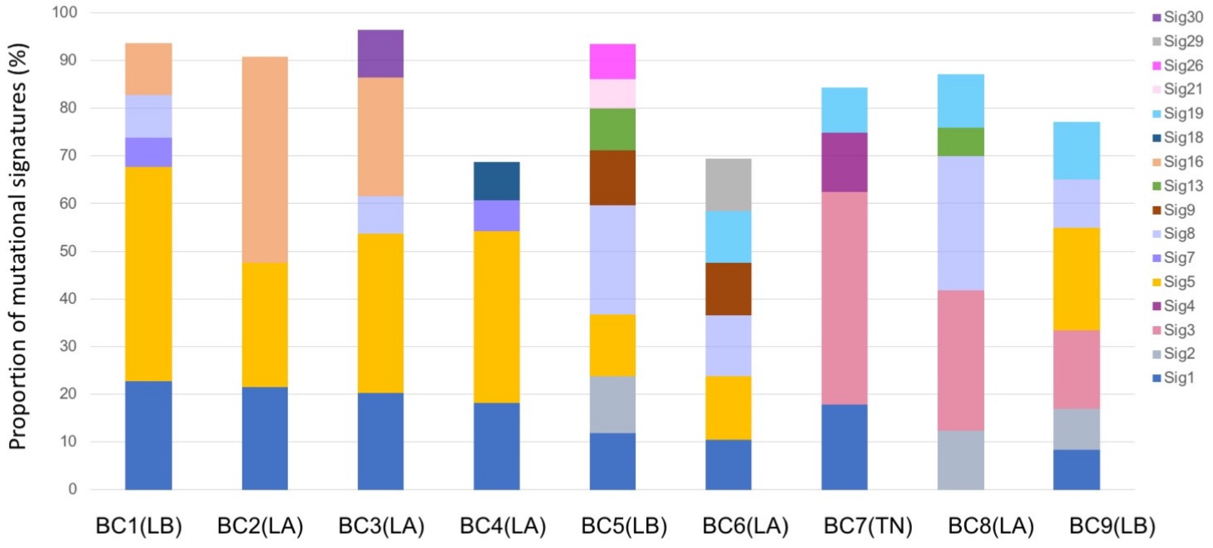

Supplementary Figure 3. Mutational signatures of 11 sporadic breast cancers lacking *NTHL1* germline variants.

One tumour (BC3) demonstrated a minor fraction of signature 30. LB, luminal B breast cancer; LA luminal A breast cancer; TN, triple-negative breast cancer.

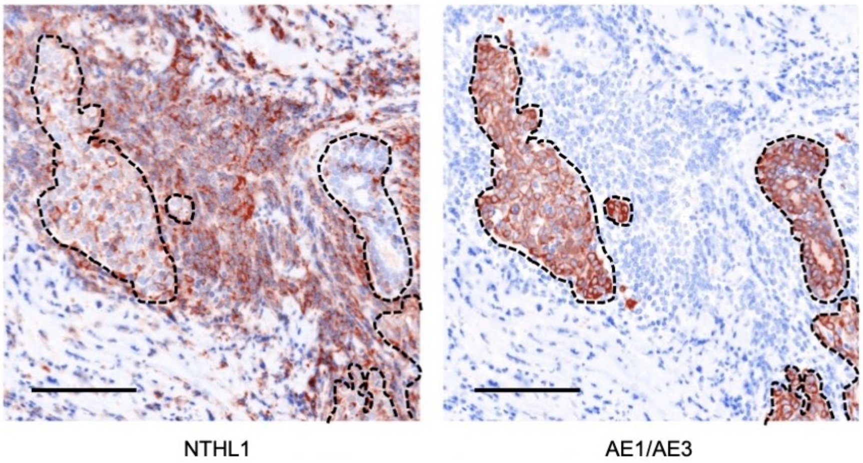

Supplementary Figure 4. NTHL1 staining in breast cancer cells and the surrounding stroma and immune cells in an *NTHL1*-het breast tumour.

Left: NTHL1 staining. NTHL1: brown color; DIPI: blue color; right: AE1/AE3 staining served as a cancer cell marker. AE1/AE3: brown color; DIPI: blue color. The fluorescence signal was converted as colorimetric pattern for display. Scale bar = 100 µm.

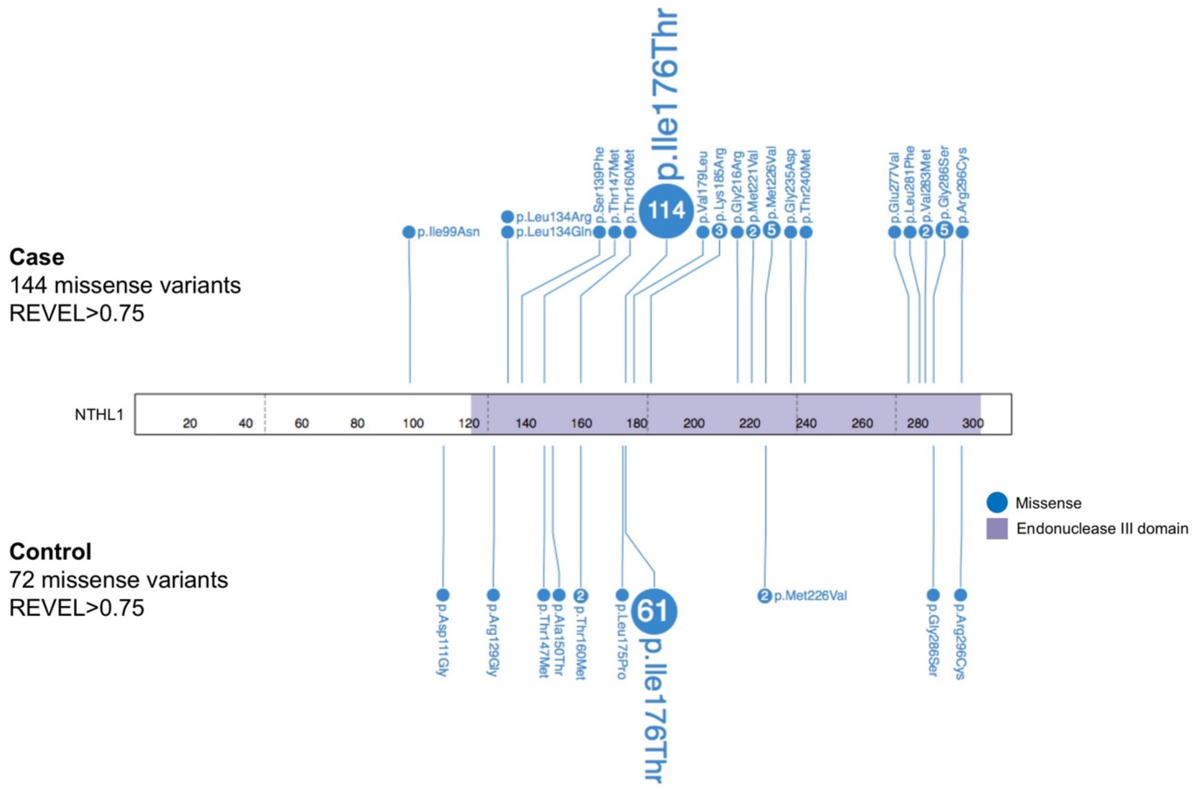

Supplementary Figure 5. Lollipop plot of missense variants with a REVEL score > 0.75 identified in 27,421 cases and 19,759 controls.
