# Supplementary material for: Evaluation of the association of heterozygous germline variants in NTHL1 with breast cancer predisposition: an international multi-center study of 47,180 subjects
Source: NPJ Breast Cancer. 2021 May 12;7:52. doi: 10.1038/s41523-021-00255-3 (PMC8115524; doi:10.1038/s41523-021-00255-3)
Supplement: Supplementary file 2 — Reporting Summary [file 41523_2021_255_MOESM2_ESM.pdf]

nature research

Corresponding author(s): Ian Campbell

Last updated by author(s): Jan 11, 2021

# Reporting Summary

Nature Research wishes to improve the reproducibility of the work that we publish. This form provides structure for consistency and transparency in reporting. For further information on Nature Research policies, see our Editorial Policies and the Editorial Policy Checklist.

## Statistics

For all statistical analyses, confirm that the following items are present in the figure legend, table legend, main text, or Methods section.

| n/a | Confirmed | |
|---|---|---|
| ☐ | ☒ | The exact sample size ($n$) for each experimental group/condition, given as a discrete number and unit of measurement |
| ☐ | ☒ | A statement on whether measurements were taken from distinct samples or whether the same sample was measured repeatedly |
| ☐ | ☒ | The statistical test(s) used AND whether they are one- or two-sided<br>*Only common tests should be described solely by name; describe more complex techniques in the Methods section.* |
| ☐ | ☒ | A description of all covariates tested |
| ☐ | ☒ | A description of any assumptions or corrections, such as tests of normality and adjustment for multiple comparisons |
| ☐ | ☒ | A full description of the statistical parameters including central tendency (e.g. means) or other basic estimates (e.g. regression coefficient) AND variation (e.g. standard deviation) or associated estimates of uncertainty (e.g. confidence intervals) |
| ☐ | ☒ | For null hypothesis testing, the test statistic (e.g. $F$, $t$, $r$) with confidence intervals, effect sizes, degrees of freedom and $P$ value noted<br>*Give P values as exact values whenever suitable.* |
| ☒ | ☐ | For Bayesian analysis, information on the choice of priors and Markov chain Monte Carlo settings |
| ☐ | ☒ | For hierarchical and complex designs, identification of the appropriate level for tests and full reporting of outcomes |
| ☐ | ☒ | Estimates of effect sizes (e.g. Cohen's $d$, Pearson's $r$), indicating how they were calculated |

*Our web collection on statistics for biologists contains articles on many of the points above.*

## Software and code

Policy information about availability of computer code

| | |
|---|---|
| Data collection | PerkinElmer Vectra Automated Multispectral Imaging System |
| Data analysis | GraphPad Prism version 7.00 (California USA); R package "meta ", 4.15-1; R version 3.6.1. |

For manuscripts utilizing custom algorithms or software that are central to the research but not yet described in published literature, software must be made available to editors and reviewers. We strongly encourage code deposition in a community repository (e.g. GitHub). See the Nature Research guidelines for submitting code & software for further information.

## Data

Policy information about availability of data

All manuscripts must include a data availability statement. This statement should provide the following information, where applicable:

- Accession codes, unique identifiers, or web links for publicly available datasets
- A list of figures that have associated raw data
- A description of any restrictions on data availability

All sequencing data are deposited to European Genome-phenome Archive which are available upon request to corresponding author

# Field-specific reporting

Please select the one below that is the best fit for your research. If you are not sure, read the appropriate sections before making your selection.

☒ Life sciences ☐ Behavioural & social sciences ☐ Ecological, evolutionary & environmental sciences

For a reference copy of the document with all sections, see nature.com/documents/nr-reporting-summary-flat.pdf

# Life sciences study design

All studies must disclose on these points even when the disclosure is negative.

| | |
|---|---|
| Sample size | The sample size was the maximum number of cases and controls available for analysis. |
| Data exclusions | Cases were excluded if a mutation in BRCA1 or BRCA2 was detected. |
| Replication | not applicable |
| Randomization | Cases and controls were not randomized. Cases were women with a personal and family history of breast cancer. Controls were cancer free women from the general population. |
| Blinding | There was no blinding. The data generated was sequencing data to determine the presence or absence of a mutation. The mutation calling was performed by an automated pipeline for both cases and controls. This is not influenced by interpretation biases. |

# Reporting for specific materials, systems and methods

We require information from authors about some types of materials, experimental systems and methods used in many studies. Here, indicate whether each material, system or method listed is relevant to your study. If you are not sure if a list item applies to your research, read the appropriate section before selecting a response.

## Materials & experimental systems

| n/a | Involved in the study | |
|---|---|---|
| ☐ | ☒ | Antibodies |
| ☒ | ☐ | Eukaryotic cell lines |
| ☒ | ☐ | Palaeontology and archaeology |
| ☒ | ☐ | Animals and other organisms |
| ☐ | ☒ | Human research participants |
| ☒ | ☐ | Clinical data |
| ☒ | ☐ | Dual use research of concern |

## Methods

| n/a | Involved in the study | |
|---|---|---|
| ☒ | ☐ | ChIP-seq |
| ☒ | ☐ | Flow cytometry |
| ☒ | ☐ | MRI-based neuroimaging |

## Antibodies

| | |
|---|---|
| Antibodies used | NTHL1 antibody (Abcam, Branford, Connecticut, USA), AE1/AE3 antibody (multi-cytokeratin antibody, Leica Biosystems, Wetzlar, Germany), HRP-labelled anti-rabbit antibody (PerkinElmer Waltham, Massachusetts, USA) and anti-mouse antibody (PerkinElmer, Waltham, Massachusetts, USA). |
| Validation | Antibodies have been validated by the manufacturing companies. |

## Human research participants

Policy information about studies involving human research participants

| | |
|---|---|
| Population characteristics | The cases study participants were all female with a personal and family history of breast cancer and over the age of 18. The controls were women in the Lifepool cohort (www.lifepool.org) who were above 40 years old and cancer-free as of May 2016. |
| Recruitment | The cases were recruited through familial cancer centres in Australia and the controls were recruited through breast screen Victoria. |
| Ethics oversight | the Human Research Ethics Committee at the Peter MacCallum Cancer Centre |

Note that full information on the approval of the study protocol must also be provided in the manuscript.
